# mRNA lipid nanoparticle vaccines: current status, challenges and future prospects

**DOI:** 10.1186/s43556-026-00600-7

**Published:** 2026-09-28

**Authors:** Zhineng Wu, Jinghu Lou, Yi Cheng, Meng Li, Zengming Wang, Nan Liu, Xiang Gao, Aiping Zheng, Hui Zhang

**Affiliations:** Academy of Military Medical Sciences, Beijing, 100850 China

**Keywords:** MRNA vaccines, Lipid nanoparticles, Stability, AI-assisted design, Thermostable formulations, Infectious diseases, Cancer immunotherapy

## Abstract

mRNA vaccines have become a clinically validated vaccine platform, as demonstrated by the success of COVID-19 vaccines such as Comirnaty and Spikevax, owing to their rapid design, manufacturing scalability, and capacity to induce in situ antigen expression. A key factor underlying this success is the lipid nanoparticle (LNP) delivery system, which protects mRNA from degradation and promotes efficient cellular uptake and cytoplasmic delivery, thereby enabling the full potential of mRNA technology. The continued advancement of mRNA-LNP vaccines requires integrated optimization of mRNA design, LNP composition, and delivery strategies to achieve improved stability, efficient intracellular delivery, and balanced immune responses. Despite remarkable progress, challenges related to formulation stability, long-term storage stability, safety and reactogenicity concerns, and durability of immune protection continue to hinder the broader application of mRNA-LNP vaccine platforms. This review provides an integrated overview of recent advances in mRNA-LNP vaccines, covering mRNA molecular engineering, LNP composition and delivery mechanisms, immune responses, clinical progress, and current developmental challenges. Furthermore, emerging strategies, including thermostable formulations, next-generation LNPs with improved targeting capability, emerging RNA platforms, and artificial intelligence-assisted optimization of RNA sequences and lipid materials, are discussed. By summarizing current achievements and future opportunities, this review highlights key principles guiding the rational design of safer, more stable, and more precise mRNA-LNP vaccine platforms and provides insights into accelerating their clinical translation.

## Introduction

Vaccination remains one of the most effective public-health interventions, preventing millions of deaths each year and playing a central role in the control of infectious diseases [1, 2]. Conventional vaccine platforms—including live-attenuated, inactivated, and subunit vaccines—have achieved remarkable success in global immunization programs. However, these approaches often involve lengthy development cycles, reliance on cell-based manufacturing, biosafety concerns associated with pathogen handling, and limited responsiveness to emerging pandemic threats [3–6]. The COVID-19 pandemic highlighted these limitations and emphasized the urgent need for vaccine technologies that enable rapid design, platform versatility, and scalable production [7]. In this context, messenger RNA (mRNA) vaccines have emerged as a transformative platform capable of accelerating vaccine development and enabling precise antigen expression [8, 9].

Since the discovery of mRNA in 1961, its therapeutic potential has gradually been explored, with early demonstrations of in vitro-transcribed mRNA expression in animal models emerging in the 1990 s [10, 11]. However, the clinical application of mRNA was long hindered by its intrinsic instability and inefficient intracellular delivery. The development of lipid-based delivery systems represented a pivotal breakthrough in overcoming these barriers [12]. Early liposome formulations explored in the 1970 s demonstrated the feasibility of nucleic-acid delivery but were limited by poor stability and low delivery efficiency. A major advance occurred in the 2010 s with the optimization of ionizable lipid chemistry and the refinement of four-component lipid nanoparticles (LNPs), which enabled efficient protection and intracellular delivery of mRNA and laid the foundation for clinically viable mRNA vaccines [13]. This technological progress was validated during the COVID-19 pandemic, when the LNP-formulated mRNA vaccines BNT162b2 (Pfizer/BioNTech) and mRNA-1273 (Moderna) received emergency use authorization and demonstrated high efficacy in preventing SARS-CoV-2 infection and lessening the severity of the symptoms [14, 15]. More recently, Moderna's RSV vaccine (mRNA-1345) received FDA approval in 2024 for preventing respiratory syncytial virus–associated lower respiratory tract disease in adults aged ≥ 60 years, further confirming the clinical viability of the mRNA-LNP platform [16–18].

Despite these advances, early approved mRNA vaccines, including those initially deployed against COVID-19, faced critical limitations, including formulation instability and stringent cold-chain requirements (e.g., storage at − 80 °C or − 20 °C), waning immunity, and the risk of aberrant inflammatory responses that compromise their overall efficacy and safety [19–21]. Subsequent iterations, such as Spikevax, have since improved storage profiles, now supporting refrigeration for up to one month and long-term storage at − 20 °C. Addressing the remaining challenges is essential for next-generation mRNA platforms to achieve more durable protection, broader deployability, and equitable global distribution [22, 23].

This review provides a comprehensive overview of the mRNA-LNP vaccine platform. It first discusses the structural design and immunological mechanisms underlying mRNA-LNP vaccines, including mRNA architecture and lipid nanoparticle delivery systems. It then summarizes the current clinical development and applications of mRNA-LNP vaccines across multiple disease areas. Next, key challenges limiting broader implementation—such as stability constraints, delivery barriers, and safety considerations—are examined. Finally, emerging innovations and future directions aimed at improving the efficacy, stability, and accessibility of mRNA-LNP vaccines are discussed. Collectively, this review aims to provide insights that may guide the rational design and future development of mRNA-LNP vaccine technologies.

## Composition and mechanism of mRNA-LNP vaccines

The efficacy of mRNA-LNP vaccines derives from the coordinated design of two fundamental components: the mRNA construct and LNP carrier. The mRNA encodes the antigen and defines immunogenic potential, whereas the LNP protects the transcript and mediates its efficient cellular delivery. Together, they determine translation efficiency, molecular stability, and the magnitude of immune activation [24]. This section outlines current advances in the design of mRNA structural elements (cap, UTRs, ORF, and poly(A) tail), next-generation RNA formats, and LNP composition and delivery mechanisms, highlighting how molecular architecture underpins effective immune responses.

### The mRNA component

mRNA vaccines rely on synthetic mRNA molecules engineered to mimic naturally occurring mRNA [25]. These molecules are designed to encode specific antigenic proteins derived from the target pathogen, which are typically viral surface proteins. In vitro–transcribed mRNAs consist of five major structural elements: a 5' cap, a 5' untranslated region (UTR), an open reading frame (ORF), a 3' UTR, and a poly(A) tail [26].

#### Structural elements of mRNA

The 5' cap is a modified nucleotide structure located at the 5' end of mature mRNA. It contains a guanine nucleotide linked to the mRNA via a triphosphate bridge and further methylated at the N7 position of guanine and/or the 2'-hydroxyl group of the first transcribed nucleotide [27]. This cap protects mRNA from exonuclease degradation, regulates pre-mRNA splicing, initiates translation, enhances translation efficiency, and facilitates mRNA export from the nucleus to the cytoplasm [28]. Post-transcriptional modifications, including 2'-O-methylation of the ribose of the first nucleotide (Cap 1), further improve translation and protein yield. In industrial in vitro transcription (IVT) processes, cap analogs are commonly employed. Five major types are frequently used: m^7^GpppG (cap 0), m^7^GpppGm (cap 1), m^7^GpppGm₂ (cap 2), antireverse cap analogs (ARCA), and β-thio-containing antireverse cap analog (β-S-ARCA) [29].

The UTRs of mRNA include the 5'-UTR and 3'-UTR flanking the coding sequence and play critical roles in the regulation of gene expression. While the coding sequence (CDS) determines the amino acid composition of the protein, the UTRs indirectly govern protein yield and function by affecting mRNA stability, translation efficiency, subcellular localization, and protein expression levels [30].

The 5'-UTR primarily controls translation initiation [31]. Its sequence, length, and secondary structure strongly influence ribosome binding and scanning efficiency. A stable Cap 1 structure and optimized UTR length enhance translation and protect against exonuclease degradation. Conversely, overly long or GC-rich 5'-UTRs may form complex secondary structures that impede ribosome access and reduce translation. Alternative splicing can also reshape the UTR and thereby modulate translation efficiency [32]. Highly efficient 5'-UTRs used in mRNA design are often derived from human α- or β-globin genes, which promote strong translation initiation and protein expression. In addition, internal ribosome entry sites (IRESs) can enable cap-independent translation under specific conditions, further enhancing mRNA stability and adaptability [33].

The 3'-UTR plays a key role in mRNA stability, localization, and post-transcriptional regulation [34]. By binding to RNA-binding proteins (RBPs) and microRNAs, it controls mRNA degradation rates and half-life. Moderately extended 3'-UTRs can increase stability and protein production, whereas excessively long or AU-rich regions often reduce half-life. Efficient 3'-UTRs frequently employ sequences from human α- or β-globin genes; for instance, the double β-globin repeat (2hBg) has become a classic IVT-mRNA motif that greatly enhances protein expression. Furthermore, the 3'-UTR contributes to protein–protein interaction networks, affecting protein modification, conformation, and complex assembly [35]. Overall, the UTRs serve as critical post-transcriptional regulatory elements and central targets for synthetic biology and mRNA-based drug design, where sequence optimization is essential for achieving efficient and stable protein expression [36].

ORF is the core region of the mRNA that is translated into protein, beginning with a start codon and ending with a stop codon. Its sequence determines the structure and function of the encoded protein [37]. In mRNA vaccines, the ORF is a key determinant of immunogenicity and translation efficiency. Optimizing the coding sequence improves correct protein folding and expression levels [38]. Codon optimization, through matching codon usage to human translation preferences, avoiding rare codons, and moderately increasing GC content, enhances mRNA stability and translation. Moreover, introducing modified nucleosides (e.g., N^1^-methyl-pseudouridine or 5-methyl-cytidine) during IVT can markedly boost stability and translation while attenuating innate immune activation, thereby improving vaccine efficacy and safety [39]. In addition, functional peptide sequences encoded within the ORF, such as signal peptides or intracellular trafficking and antigen-processing motifs, can be incorporated to regulate antigen localization and presentation. These sequences are translated as part of the expressed protein rather than being included as exogenous peptides in the formulation, thereby promoting specific CD4⁺ and CD8⁺ T-cell responses. In summary, a well-engineered ORF enables robust, stable, and highly immunogenic protein expression, constituting the molecular foundation for successful mRNA vaccines and therapeutic mRNAs [40].

The poly(A) tail is a stretch of approximately 10–250 adenosine residues added to the 3' end of mRNA during post-transcriptional processing and is an essential structural component of mRNA [41]. It stabilizes the transcript, promotes nuclear export to the cytoplasm, and enhances translation efficiency. By interacting with poly(A)-binding proteins (PABPs), the tail facilitates the formation of a closed-loop structure that improves ribosome recruitment and translation efficiency. Tail length (typically ≥ 30–40 adenosines) directly affects mRNA stability and protein output by preventing exonuclease degradation [42]. In mRNA vaccines such as Moderna’s mRNA-1273 and Pfizer-BioNTech’s BNT162b2, poly(A) tails are meticulously designed to maximize stability and translation, using either uniform or segmented structures with specific linker sequences. Beyond conventional linear poly(A)-tail engineering, emerging topological engineering strategies, such as chemically modified branched poly(A) tails, have recently been shown to enhance mRNA translation capacity and prolong protein expression in vitro and in vivo, highlighting new opportunities for optimizing mRNA therapeutics [43]. Studies also indicate that introducing non-adenosine residues (e.g., cytidine) at the 3' end can further prolong protein expression [44]. The poly(A) tail can be synthesized enzymatically post-transcriptionally or co-transcriptionally, and its proper design is crucial to ensuring mRNA functionality and therapeutic performance.

#### mRNA architecture

##### Non-replicating mRNA

Non-replicating mRNA vaccines encode only the target antigen and do not contain genes for replication enzymes. While the mRNA constructs may vary in size depending on the target antigen, they are generally characterized by a simpler design compared to traditional vaccine platforms, offering high specificity in immune responses [45]. These vaccines rely on LNPs for delivery into host cells, where the mRNA is translated in the cytoplasm to produce the antigen, thereby activating both humoral and cellular immune responses. Compared with self-amplifying mRNA, the expression of non-replicating mRNA is limited, requiring higher doses to achieve the desired immunization effect, or necessitating comprehensive optimization to elicit effective immune responses at reduced doses.

##### Self-Amplifying RNA (saRNA)

SaRNA technology represents a next-generation RNA platform capable of amplifying antigen expression. Compared with conventional linear nonreplicating mRNA, saRNA or self-amplifying mRNA incorporates additional elements into the linear mRNA backbone that encode both replicase components and the antigen sequence [46]. These replicase components comprise four nonstructural proteins (nsP1, nsP2, nsP3, and nsP4) derived from alphaviruses, enabling self-amplifying mRNA molecules not only to self replicate but also to generate large quantities of target proteins. As a result, saRNA transcripts are substantially longer than conventional mRNA, with typical vaccine mRNAs ranging from approximately 1,000 to 4,000 nt, whereas saRNA commonly spans approximately 9,000 to 12,000 nt, depending on UTR design and poly(A) length. Replication intermediates such as double-stranded RNA accumulate within cells, supporting potent protein expression and strong immunogenicity even at lower doses. To mitigate replication associated innate immune activation and improve expression yield, several molecular engineering approaches have been explored, such as splitting the replicase into two transcripts, incorporating modified nucleosides, or introducing stabilizing mutations into replicase domains to reduce double-stranded RNA formation and suppress innate immune recognition. These strategies have demonstrated improved transfection efficiency or reduced inflammatory responses across multiple experimental models [47, 48]. Despite such progress, the intrinsic self-amplifying nature of saRNA, while conferring marked dose sparing and sustained expression advantages, can also trigger innate immune activation that suppresses translation or induces inflammation, underscoring the need for continued refinement [49]. Consequently, successful clinical translation of saRNA will depend not only on further optimization of sequence and nucleotide modifications but also on the development of low toxicity, high efficiency delivery systems together with rigorous safety evaluations [50, 51].

##### Circular RNA (circRNA)

Circular RNAs are covalently closed single-stranded RNA molecules that lack both a 5′ cap and a 3′ poly(A) tail. Unlike linear mRNAs, circRNAs are primarily translated through cap-independent mechanisms, most commonly via internal ribosome entry sites (IRESs). Their closed-loop structure confers intrinsic resistance to exonuclease-mediated degradation, resulting in increased intracellular stability and prolonged half-life, which may support more sustained antigen expression and enhanced immune responses [52]. These characteristics have made circRNAs an attractive platform for RNA-based vaccines and therapeutics. For example, Qu et al. developed an IRES-driven circRNA-based SARS-CoV-2 vaccine that induced higher antigen expression and stronger immune responses, highlighting the potential of circRNA as an alternative to conventional mRNA platforms [53]. Additional studies have further demonstrated the promise of circRNA in vaccine development and cancer immunotherapy, including strong protective efficacy in monkeypox virus models and immune responses comparable to or greater than those induced by self-amplifying mRNA in cancer immunization settings [54]. Moreover, circRNA formulations have shown reduced inflammatory reactivity, improved storage stability, and prolonged protein expression both in vitro and in vivo [55, 56]. Lyophilized circRNA-LNP formulations have also exhibited sustained stability and retained immunogenicity for up to 24 weeks at 4 °C in animal studies [57]. However, several challenges remain, including dependence on IRES activity, limited cyclization efficiency, and difficulties in large-scale production and purification [58]. To facilitate circRNA design and functional evaluation, bioinformatic resources such as circBank [59] and CICADA [60] have been developed and are increasingly used in circRNA research. Overall, these findings support circRNA as a promising next-generation platform for RNA-based vaccines and therapeutics [61].

### The LNP delivery system

Naked mRNA is inherently unstable and highly susceptible to degradation by extracellular ribonucleases (RNases), and it exhibits extremely low efficiency in passive transmembrane transport [62]. A variety of non-viral delivery approaches, including physical methods, protamine-based systems, polymer complexes, and dendritic cell loading, have been explored to enhance mRNA stability and cellular uptake. Although some of these strategies showed encouraging preclinical results, many have faced limitations related to delivery efficiency, scalability, or practical application. Importantly, polymer-based materials remain an active and high-interest area of research in drug and nucleic acid delivery. In this context, the development of LNP systems containing ionizable lipids in the 2010 s represented a major advance and enabled the broad clinical translation of mRNA therapeutics and vaccines [63].

Lipid nanoparticles are typically formulated with ionizable lipids, cholesterol, phospholipids, and polyethylene glycol (PEG) lipids. With a diameter around 100 nm, these nanoparticles efficiently encapsulate and protect mRNA and facilitate their systemic delivery to target cells via the bloodstream. Owing to their proven biocompatibility, stability, and exceptional capacity to shield mRNA from degradation, LNPs have become the dominant delivery platform for mRNA vaccines [64, 65].

Ionizable lipids are pivotal molecules in nucleic acid delivery systems, particularly in LNP carriers for mRNA vaccine [66]. Their functionality relies on a pH-dependent charge-switching ability, which allows them to achieve an optimal balance between in vivo delivery efficiency and safety [67].

Typically, ionizable lipids consist of three structural units: a polar amine headgroup, a linker, and hydrophobic tails. The amine head determines the pKa and protonation capacity, and it can exist in monoamine or polyamine forms. The linker (e.g., ester or amide bonds) influences the stability and biodegradability of the lipid, while the hydrophobic tails regulate membrane fluidity, lipophilicity, and fusion capability, which are factors that critically affect mRNA encapsulation efficiency and endosomal escape [68]. Representative ionizable lipids used in clinically approved mRNA vaccines include SM-102 and ALC-0315. SM-102 is employed in Moderna's mRNA-1273 vaccine, whereas ALC-0315 is used in Pfizer/BioNTech's BNT162b2 vaccine. Although structurally similar, these two lipids differ slightly in the branching and length of their hydrophobic tails; the longer tails of SM-102 contribute to enhanced LNP stability and membrane fusion. Over the past two decades, extensive efforts have driven the development and structural evolution of a broad range of ionizable lipids, with structure–function studies increasingly guiding the design of new lipid structures for nucleic acid delivery [69, 70].

Phospholipids serve as essential helper components in LNPs, primarily contributing to structural stabilization, mRNA encapsulation, and delivery efficiency [71]. Phospholipids such as DSPC and DOPE modulate LNP stability and membrane fusion through their distinct molecular geometries. DSPC contains saturated long-chain fatty acids and has a cylindrical shape, providing high physical stability and rigidity under physiological conditions; it is widely used in clinically approved mRNA vaccines, such as Moderna’s mRNA-1273 and BioNTech/Pfizer’s BNT162b2 [72]. In contrast, DOPE contains unsaturated fatty acid chains and has a cone-like structure. The choice of phospholipid can influence LNP stability, biodistribution, and delivery potency in a formulation-dependent manner [73]. Recent advances have introduced novel functional phospholipids, such as alcohol-soluble phosphatidylserine derivatives that enhance targeting to the spleen and lymphoid tissues, and pH-responsive zwitterionic ionizable phospholipids (e.g., 9A1P9) that adopt a cone-shaped structure in acidic endosomal environments, inducing membrane phase transitions and significantly improving mRNA release [74]. Overall, phospholipids play a crucial role in maintaining LNP physical stability, promoting membrane fusion, and controlling tissue targeting.

Cholesterol is a naturally occurring hydrophobic lipid and an essential component of biological membranes. It maintains membrane structural integrity and function by modulating membrane fluidity, thickness, rigidity, and compressibility. Within lipid nanoparticles, cholesterol serves as a key helper lipid that fills inter-lipid spaces, enhances membrane cohesion, and promotes fusion with endosomal membranes. Consequently, it improves particle stability, extends circulation half-life, and increases mRNA delivery efficiency [75]. Studies have shown that cholesterol derivatives can further influence LNP morphology, lamellar organization, and biodistribution. For instance, complete replacement of cholesterol with plant sterols such as β-sitosterol has been shown to enhance LNP transfection performance in specific experimental systems [76]. Cholesterol thus imparts excellent structural stability and biocompatibility to LNPs, while also playing a critical regulatory role in gene and drug delivery.

PEG-lipids are essential components of LNP formulations but are typically incorporated in relatively small quantities, often at approximately 0.5–1.5 mol% depending on the formulation design. In LNP systems, PEG-lipids form a steric barrier on the particle surface that limits protein adsorption, aggregation, and nonspecific interactions while enhancing colloidal stability. Structural parameters such as PEG chain length and lipid anchor composition strongly influence particle size, surface characteristics, and formulation stability [77]. For example, DMG-PEG2000 is widely used in clinically approved mRNA products, where it helps maintain dispersion stability and ensures reproducible physicochemical characteristics during manufacturing and storage [78]. For mucosal and respiratory LNP delivery, higher PEG-lipid contents are often explored to improve particle stability and mucus penetration, although excessive PEGylation can reduce transfection efficiency [79]. In addition, alternative hydrophilic polymers such as polysarcosine have also been explored as potential substitutes for PEG in nanoparticle formulations [80].

Mucosal delivery represents an emerging research direction for mRNA-LNP vaccine development. Compared with conventional systemic administration, mucosal delivery requires LNPs to overcome mucus barriers and achieve efficient delivery at mucosal surfaces. Recent studies have demonstrated that PEG-lipid optimization plays an important role in respiratory and mucosal LNP delivery by affecting colloidal stability and delivery performance [81]. In addition, PEG-lipid modification strategies have been explored to improve the mucosal immune responses of intranasally administered mRNA-LNP vaccines [82]. However, excessive PEGylation may reduce cellular interaction and transfection efficiency, highlighting the importance of balancing PEG density and vaccine performance.

### Mechanism of action: from injection to immune response

Traditional vaccines (e.g., inactivated, subunit, or conjugate vaccines) are typically composed of the pathogen-specific antigens themselves, combined with adjuvants to enhance the induction of a specific adaptive immune response. In contrast, mRNA-LNP vaccines can stimulate innate immune pathways through both the mRNA and the lipid nanoparticle components; however, excessive mRNA sensing may suppress antigen expression, and optimized LNP formulations substantially contribute to the adjuvant effect [83, 84].

**Fig. 1:**
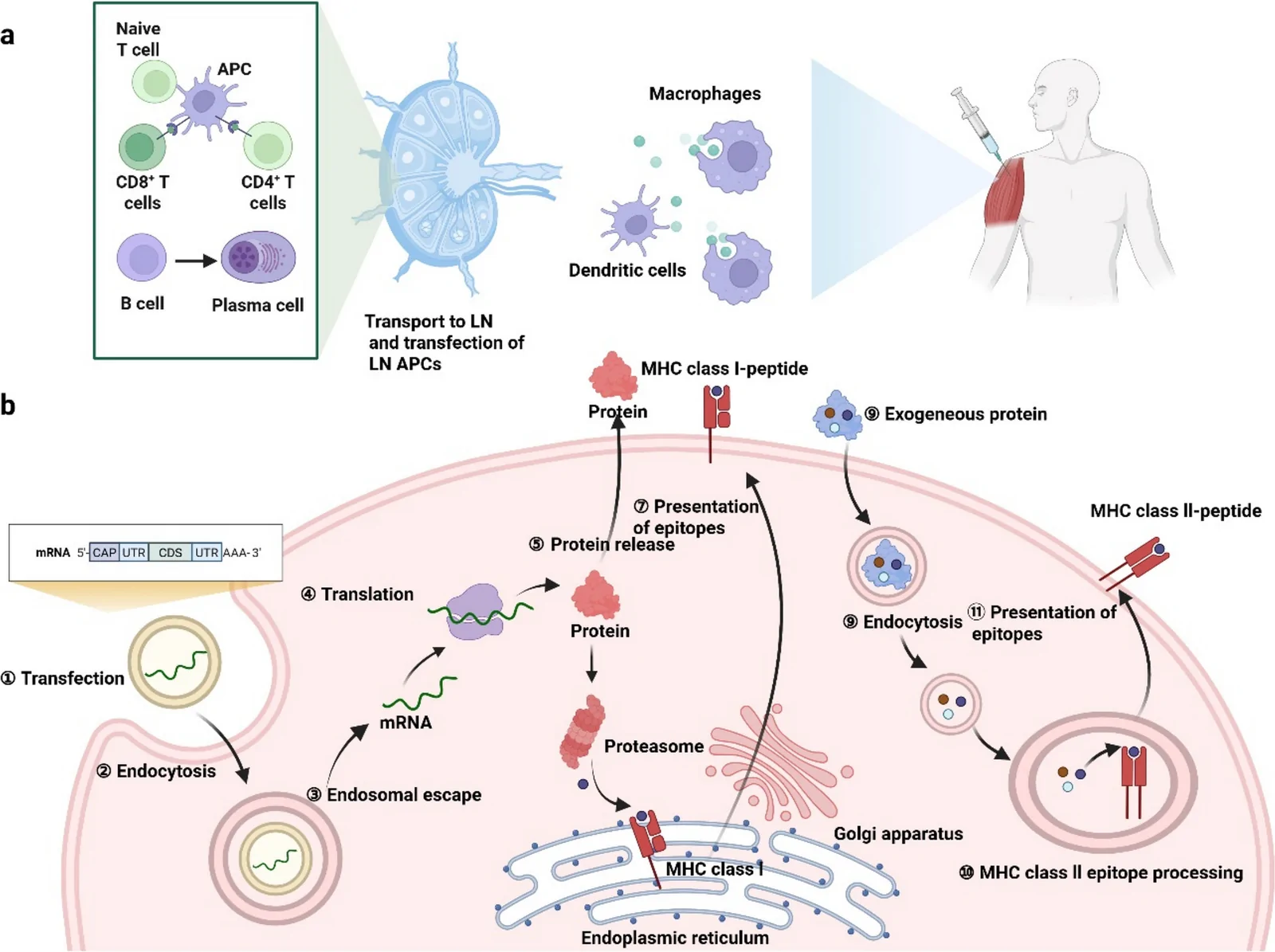
Mechanism of Intramuscular mRNA-LNP Vaccine Action. **a** illustrates the distribution and mechanism of action of mRNA–lipid nanoparticle (mRNA-LNP) vaccines at the intramuscular injection site. Following administration, mRNA-LNPs can enter muscle cells and transfect tissue-resident antigen-presenting cells (APCs), thereby initiating a localized immune response. Simultaneously, a portion of the mRNA-LNPs drains into lymph nodes (LNs) via lymphatic circulation, where they are taken up and expressed by lymph node–resident cells, leading to further activation of T and B cell responses. **b** The principal processes by which mRNA-LNP vaccines elicit adaptive immune responses are as follows. First, in vitro–transcribed mRNA is encapsulated within lipid nanoparticles to enable stable delivery. Subsequently, mRNA-LNPs enter host cells through lipid-mediated membrane fusion or endocytosis. Once internalized, mRNA escapes from endosomes into the cytoplasm. The released mRNA is then translated by host ribosomes into antigenic proteins, which undergo proteasomal degradation. The resulting peptide fragments are loaded onto major histocompatibility complex class I (MHC I) molecules and presented on the cell surface to activate CD8⁺ cytotoxic T lymphocytes. Meanwhile, a portion of exogenous antigens can also be presented via the MHC class II pathway, promoting CD4⁺ helper T cell activation. Additionally, antigens released into the extracellular environment can be recognized by B cells, triggering their differentiation and maturation, and ultimately leading to the establishment of a humoral immune response

**Fig. 2:**
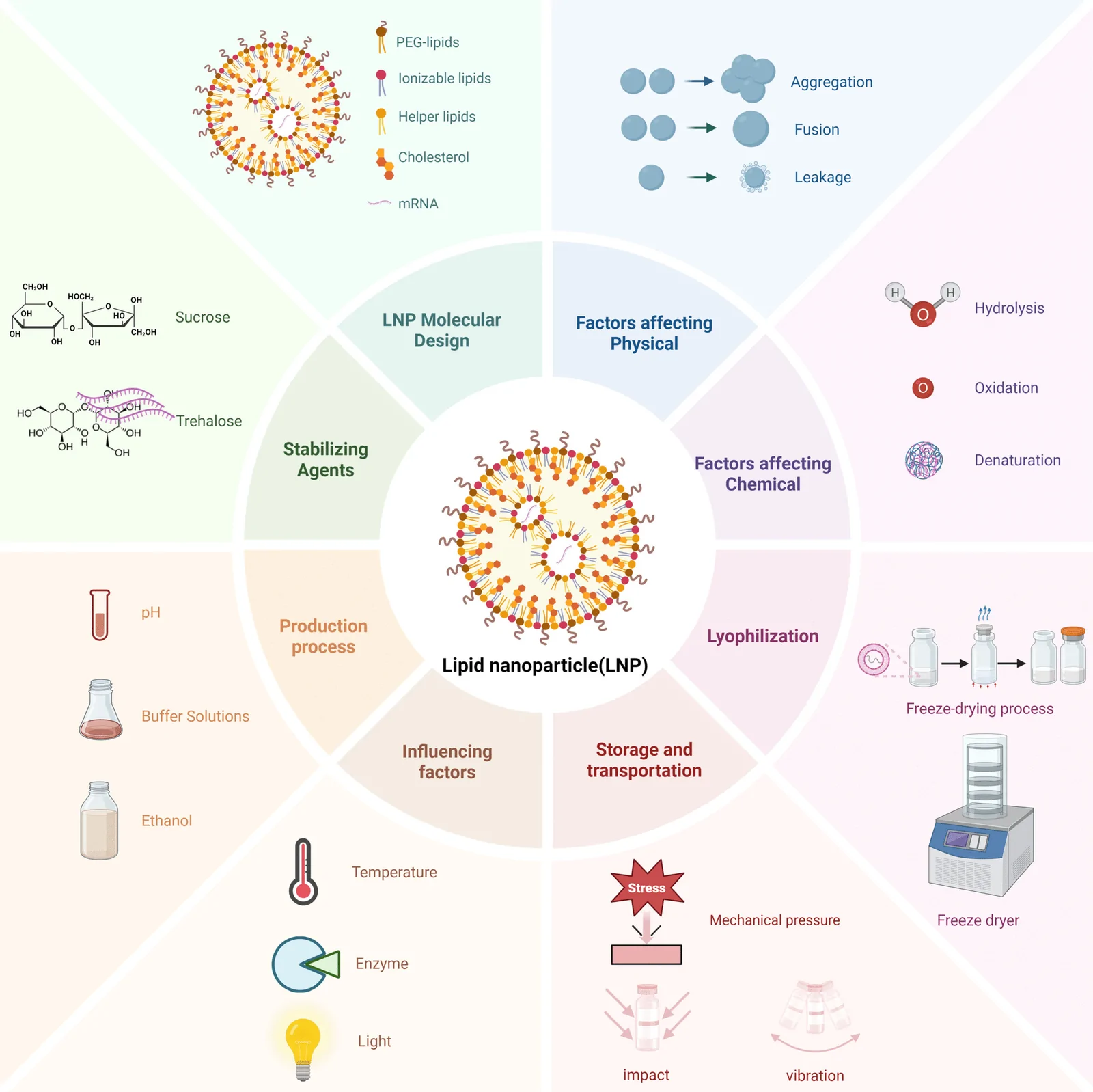
Determinants of LNP–mRNA stability across design, manufacturing, lyophilization, and storage-transportation stages. This schematic provides a comprehensive overview of the multi-layered factors influencing the stability and degradation of lipid nanoparticle (LNP)–based mRNA formulations. At the center is a representative four-component LNP encapsulating mRNA, surrounded by key domains that contribute to stability control: molecular and formulation design (PEG-lipids, ionizable lipids, helper lipids, cholesterol, and mRNA); physical instabilities (aggregation, fusion, leakage); chemical instabilities (hydrolysis, oxidation, denaturation); lyophilization processes (freeze-drying procedures and equipment); storage and transportation stresses (mechanical pressure, impact, vibration); environmental and external factors (temperature, enzymatic activity, light exposure); production process conditions (pH, buffer composition, ethanol); and stabilizing excipients (sucrose, trehalose). The figure highlights that mRNA-LNP vaccine quality and potency are collectively determined by an integrated interplay of formulation design, processing parameters, and environmental control throughout the entire life cycle—from synthesis to final distribution

Following intramuscular injection, mRNA-LNP vaccines initiate immune responses through multiple pathways (Fig. 1). On one hand, they transfect local muscle cells and keratinocytes; on the other, they directly enter tissue-resident antigen-presenting cells (APCs), such as dendritic cells (DCs), macrophages, and Langerhans cells. Upon migration of these APCs to secondary lymphoid organs, including lymph nodes and the spleen, T cell and B cell activation is further promoted. As illustrated in Fig. 2, once delivered intramuscularly, mRNA-LNP vaccines are recognized by the host’s pattern recognition receptors (PRRs) located in endosomes and the cytoplasm and at the cell surface. In addition to RNA-sensing receptors such as endosomal TLR3 and TLR7 and cytosolic sensors including RIG-I, MDA5, NOD2, and PKR, lipid-sensing TLR4 may also be activated by the ionizable lipid component of LNPs. Activation of these pathways triggers NF-κB/IRF signaling, leading to the production of pro-inflammatory cytokines and type I interferon-associated responses. Excessive innate sensing may suppress cellular translation machinery and thereby limit antigen expression [85, 86].

The immunological mechanism of mRNA vaccines relies on their ability to transfect non-immune cells, enabling these cells to synthesize and express the encoded target antigen. The expressed antigenic proteins are subsequently degraded by the proteasome in the cytoplasm, and the resulting peptides are loaded onto major histocompatibility complex (MHC) class I molecules. This process induces CD8⁺ cytotoxic T cell responses, establishing cellular immunity against the mRNA-encoded antigen [87]. Transfection of muscle cells by mRNA vaccines can indirectly activate bone marrow–derived dendritic cells, thereby promoting the priming of CD8⁺ T cells and leading to localized immune activation at the injection site. Once transfected, these immune cells present antigen peptides via the MHC class I pathway to drive CD8⁺ T cell differentiation, while activation of APCs also engages the MHC class II pathway, further stimulating CD4⁺ helper T cells [88].

After transfection of both local immune and non-immune cells, a portion of the mRNA vaccine can drain into lymph nodes, where it interacts with monocytes as well as naïve T and B cells. Transfection of APCs within the lymph nodes not only triggers T cell responses but also facilitates B cell activation and subsequent antibody production.

## Current status and clinical success

The clinical success of mRNA-LNP vaccines has transformed RNA technology from an experimental concept into a mature medical platform. Since the approval of the first COVID-19 vaccines, mRNA therapeutics have rapidly expanded to a broad range of infectious and non-infectious diseases [89]. This section reviews the key clinical milestones achieved to date, spanning from the initial COVID-19 vaccines that established the safety and efficacy of mRNA-LNP technology to the development of next-generation vaccines targeting diverse viruses and cancers. It highlights how continuous advances in molecular design, delivery systems, and immune mechanisms are collectively driving the transition of vaccinology from prevention to therapy.

### COVID-19 vaccines

At present, eight LNP-based mRNA vaccines have been approved and authorized for human use, seven of which target COVID-19 and one targets respiratory syncytial virus (RSV). The first two mRNA vaccines to receive emergency use authorization, Comirnaty® (BNT162b2) and Spikevax® (mRNA-1273), represent milestone products of LNP delivery technology (Table 1). Both follow a two-dose prime-boost regimen, containing 30 µg and 100 µg of nucleoside-modified mRNA, respectively, administered via intramuscular injection (i.m.). The LNP formulation of Comirnaty® consists of the ionizable lipid ALC-0315, the structural lipid DSPC, cholesterol, and the PEG-lipid ALC-0519, at a molar ratio of 46.3: 9.4: 42.7: 1.6 with an N/P ratio of 6.00. The Spikevax® LNP comprises the ionizable lipid SM-102, DSPC, cholesterol, and the PEG-lipid DMG-PEG2000, with an estimated molar ratio of 50: 10: 38.5: 1.5 and an N/P ratio of 5.67.

**Table 1:** Currently approved LNP-based mRNA vaccines

Generic name	Approved indications	Administration route	Original developer	Approved regions	Approval date	References
Tozinameran (BNT162b2)	COVID-19; SARS-CoV-2 infection; Coronavirus infection	Intramuscular injection	BioNTech SE; Pfizer	United States, European Union, Japan	December 11, 2020	[90]
Elasomeran	COVID-19; SARS-CoV-2 infection; Coronavirus infection	Intramuscular injection	Moderna Inc	United States, European Union, Japan	December 18, 2020	[91]
ARCoV	COVID-19; SARS-CoV-2 infection	Intramuscular injection	Yunnan Watson Biotechnology Co., Ltd.; Suzhou Aibo Biological Technology Co., Ltd	China	October 9, 2022	[92]
SYS6006	COVID-19; SARS-CoV-2 infection	Intramuscular injection	CSPC Zhongqi Pharmaceutical Technology (Shijiazhuang) Co., Ltd	China	March 22, 2023	[93]
ARCT-154	SARS-CoV-2 infection	Intramuscular injection	Arcturus Therapeutics Inc	Japan	November 27, 2023	[94]
RQ-3033	COVID-19	Intramuscular injection	Shanghai Blue Arrow Biomedical Co., Ltd.; Yunnan Watson Biotechnology Co., Ltd.; Fudan University	China	December 1, 2023	[95]
mRNA-1345	Respiratory diseases; Respiratory syncytial virus infection	Intramuscular injection	Moderna Inc	United States, European Union	May 31, 2024	[96]
mRNA-1273.712	COVID-19	Intramuscular injection	Moderna Inc	United States	August 22, 2024	[97]

Since authorization, both vaccines have continued to be refined, including: (i) revision of mRNA ORF sequences to match circulating SARS-CoV-2 variants and subvariants; (ii) further refinement of buffer composition and labeled storage conditions to improve stability and facilitate distribution; and (iii) refinement of dosing intervals and manufacturing processes. These improvements have significantly enhanced the stability, accessibility, and durability of immune protection provided by mRNA vaccines [98].

As of 2025, more than one billion doses of Comirnaty® and Spikevax® have been administered globally. Extensive clinical and real-world data consistently demonstrate their high efficacy and favorable safety profiles in preventing severe disease and death. These vaccines remain the most successful applications of mRNA technology to date. However, the emergence of viral mutations and waning population immunity have revealed certain limitations of first-generation COVID-19 mRNA vaccines. Studies indicate that both humoral and cellular immune responses decline substantially within approximately six months, accompanied by reduced neutralization activity against variants such as Omicron. Consequently, periodic booster immunizations are required to sustain protection [99].

Common mild-to-moderate adverse reactions following vaccination include local pain, fatigue, fever, and headache, while serious adverse events (SAEs) occur at extremely low frequencies. Regulatory evaluations have concluded that these risks are acceptable given the vaccines’ substantial benefits in preventing severe disease. Myocarditis and pericarditis have been identified as rare but vaccine-associated adverse events, occurring more frequently in young males. A large Nordic cohort study including 8.9 million participants reported that within 28 days following a third dose of Comirnaty® or Spikevax®, the absolute risk of myocarditis in males was approximately 0.86 and 1.95 cases per 100,000 doses, respectively, and 0.15 cases among females, with no deaths observed within 30 days. These findings confirm that the overall risk–benefit profile of mRNA vaccines remains strongly favorable. Nonetheless, since intramuscular injection does not induce mucosal immunity, current vaccines are unable to fully block viral transmission, prompting the development of next-generation RNA vaccines [100].

To address ongoing SARS-CoV-2 evolution, Pfizer/BioNTech and Moderna have introduced bivalent vaccines targeting Omicron subvariants such as BA.1, BA.4, and BA.5, which have demonstrated enhanced neutralizing activity against variants in phase III clinical trials [101]. Moderna has also developed mRNA-1283, encoding only the receptor-binding domain (RBD) and N-terminal domain (NTD), which elicits comparable or superior immune responses to mRNA-1273 while exhibiting improved stability at 2–8 °C, significantly reducing cold-chain requirements. Beyond North America, several countries have approved domestically developed mRNA vaccines. For instance, China’s CSPC Pharmaceutical Group obtained emergency use authorization from the National Medical Products Administration (NMPA) for the SYS6006 vaccine, designed as a booster against BA.2 and BA.4/5 variants, achieving 85.3% protective efficacy within 14–28 days post-vaccination [102]. In Japan, the DAICHIRONA® mRNA vaccine targeting the XBB.1.5 strain was approved in December 2023; its LNP carrier comprises the ionizable lipid T168-1857a and mRNA MAFB-7256a, with an N/P ratio of 4.57. Clinical data indicate that a two-dose 60 µg regimen elicits robust humoral immune responses [103]. In Indonesia, the Chinese-developed AWcorna (formerly ARCoV) vaccine received emergency approval due to its superior thermostability and simplified storage requirements; it remains stable for six months at 2–8 °C, and a single 15 µg dose induces strong neutralizing antibody responses.

Overall, the global deployment of first-generation COVID-19 mRNA vaccines has firmly established the safety and efficacy of RNA vaccine platforms. Nevertheless, challenges such as immune waning, cold-chain dependence, and insufficient mucosal immunity remain to be addressed. The next generation of RNA vaccine development is moving toward multivalent and broad-spectrum antigens, low-dose saRNA platforms, thermostable formulations, and combination immunization strategies; for example, the bivalent mRNA-1083 influenza, COVID-19 vaccine. As of now, more than 300 clinical trials related to COVID-19 mRNA vaccines have been registered worldwide, including 67 phase III studies and 28 completed trials, providing ongoing evidence for long-term safety and cross-protection. In the coming years, continued advancements in immunological understanding and delivery systems are expected to position mRNA vaccines as leading technologies for the prevention and control of seasonal and emerging respiratory infectious diseases [104].

### Vaccines in clinical development

Beyond COVID-19 vaccines, the application of the mRNA platform has expanded to other pathogens. On May 31, 2024, the U.S. FDA approved Moderna’s mRNA-1345 (mRESVIA®) for the prevention of respiratory syncytial virus (RSV) infection in adults aged 60 years and older, marking the first approved non–SARS-CoV-2 mRNA-LNP vaccine [105]. The vaccine encodes the prefusion F glycoprotein of RSV, and a single 50 µg intramuscular injection significantly reduced the incidence of RSV-associated lower respiratory tract disease in older adults without serious safety concerns. This approval signifies the successful extension of the mRNA vaccine platform from coronavirus prevention to broader respiratory pathogens, laying the groundwork for future clinical translation against influenza, Zika virus, rabies, and human immunodeficiency virus (HIV) (Table 2). With continued advances in clinical research and optimization of delivery systems, mRNA-LNP technology is evolving from an emergency pandemic countermeasure into a multipathogen, long-term, and iteratively adaptable vaccine platform, poised to play an increasingly important role in global public health [106].

**Table 2:** Overview of mRNA vaccines applied in clinical trials for infectious diseases (excluding COVID-19)

Disease	Drug name	Phase	Status	First posted data	National Clinical Trial identifier (NTC) number	Sponsor
Influenza	CVSQIV	I	Completed	Sep. 23, 2022	NCT05252338	CureVac
Influenza	Monovalent mRNA NA vaccine	I	Completed	Jun. 21, 2022	NCT05426174	Sanofi Pasteur
Influenza	H1aF-5928 mRNA LNP	I	Active, not recruiting	Mar. 6, 2023	NCT05755620	NIAID
Influenza	mRNA-1011.1, mRNA-1011.2, mRNA-1012.1	I/II	Completed	Apr. 24, 2023	NCT05827068	Moderna
Influenza	H3 mRNA/LNP Vaccine	I	Completed	Apr. 25, 2023	NCT05829356	Sanofi Pasteur
Influenza	mRNA-1010, mRNA-1010.4, mRNA-1010.6	II	Completed	May. 23, 2023	NCT05868382	Moderna
Influenza	DCVC H1 HA mRNA LNP	I	Recruiting	Jul. 14, 2023	NCT05945485	NIAID
Influenza	ARCT-2138	I	Active, not recruiting	Nov. 9, 2023	NCT06125691	Arcturus Therapeutics
Influenza	MRTS421, MRTS424, and MRTS429	I/II	Active, not recruiting	Apr. 12, 2024	NCT06516175	Sanofi Pasteur
Influenza	GSK4382276	II	Active, not recruiting	May. 25, 2024	NCT06431607	GlaxoSmithKline
Influenza	TIV mRNA HA + TIV mRNA NA	I/II	Recruiting	Jun. 25, 2025	NCT06744205	Sanofi Pasteur
Zika Virus	mRNA-1325	I	Completed	Jun. 9, 2017	NCT05014099	Moderna
Zika Virus	mRNA-1893	II	Completed	Jun. 8, 2021	NCT04917861	Moderna
Rabies	CV7201	I	Completed	Sep. 16, 2014	NCT02241135	CureVac
Rabies	CV7202	I	Completed	Oct. 19, 2018	NCT03713086	CureVac
HIV	mRNA-1644, mRNA-1644v2-Core	I	Active, not recruiting	Aug. 11, 2021	NCT05001373	International AIDS Vaccine Initiative
HIV	BG505 MD39.3, BG505 MD39.3 gp151, and BG505 MD39.3 gp151 CD4Ko HIV trimer mRNA vaccines	I	Active, not recruiting	Feb. 1, 2022	NCT05217641	NIAID
HIV	V3G CH848 mRNA-Tr2	I	Active, not recruiting	Jun. 15, 2023	NCT05903339	NIAID
HIV	CH505M5 N197D mRNA-gp160 and CH505 TF mRNA-gp160	I	Recruiting	Aug. 16, 2024	NCT06557785	NIAID
HIV	mRNA-1645-eODGT8, mRNA-1645-20reG28v2, mRNA-1640-N332GT5	I	Not yet recruiting	Nov. 19, 2024	NCT06694753	HIV Vaccine Trials Network

Data source: ClinicalTrials.gov

#### Monkeypox (MPXV)

The 2022 global outbreak of MPXV drew widespread attention. Due to the high antigenic similarity between MPXV and variola virus (VARV), significant cross-immunity can be achieved. Consequently, early-generation smallpox vaccines demonstrated substantial protective effects against monkeypox, with MVA-BN (Modified Vaccinia Ankara–Bavarian Nordic), a third-generation non-replicating smallpox vaccine, being the most recommended vaccination strategy during the outbreak. Building upon traditional vaccine approaches, emerging mRNA vaccine technologies have been introduced into monkeypox prevention research [107]. Using conserved epitopes from MPXV, VARV, and vaccinia virus (VACV) (such as A29/A30, A35, M1, and B6), researchers employed bioinformatics-based screening and immunogenicity prediction (antigen score 0.4656, exceeding the threshold of 0.4) to construct multiepitope vaccine candidates. Among them, MPXVac-2022 developed by Fang et al. targets five key MPXV antigens (A29L, E8L, M1R, A35R, and B6R), eliciting neutralizing antibody titers 10–100-fold higher than unvaccinated controls and significantly enhancing Th1/Th2 cytokine secretion and T-cell responses in animal models. Another quadrivalent mRNA vaccine demonstrated effective activation of MPXV-specific T-cell immunity and inhibition of viral replication. The inherent advantages of mRNA vaccines, such as platform flexibility, short manufacturing cycles, and rapid antigen sequence optimization against new variants, provide a highly adaptable and efficient approach for combating monkeypox and other orthopoxvirus diseases [108].

#### Zika Virus (ZIKV)

Zika virus, a mosquito-borne flavivirus first identified in 1947, can cause severe outcomes such as microcephaly and Guillain–Barré syndrome. Although several DNA-based, inactivated, and live-attenuated ZIKV vaccines (e.g., ZPIV, MV-ZIKA, VLA1601) have reached phase I/II clinical trials, no safe and effective vaccine is currently available. mRNA vaccines have shown remarkable potential: Moderna’s mRNA-1325 (NCT03014089) and mRNA-1893 (NCT04064905), both encoding the ZIKV prM–E protein, have completed phase I trials [109]. The geometric mean titer (GMT) of mRNA-1325 reached 10.3 at a 10 µg dose, while mRNA-1893 achieved a GMT of 454.2 at 100 µg, with immune responses persisting for 13 months and neutralizing antibody titers in animal models approximately 20 times higher than those induced by mRNA-1325 [110]. Nucleoside-modified mRNA-LNP vaccines have been shown to induce strong and durable neutralizing antibody responses with a single low-dose injection (30–50 µg) in mice and rhesus macaques, offering rapid and broad-spectrum protection. Moreover, these vaccines effectively prevented fetal infection in pregnant mouse models, highlighting their promise as leading candidates for ZIKV vaccine development [111].

#### Influenza

Influenza causes an estimated 290,000–650,000 deaths worldwide each year. Among the four influenza virus types (A, B, C, and D), type A viruses are the most prone to interspecies transmission and rapid mutation through “antigenic drift” and “antigenic shift,” leading to vaccine effectiveness ranging only from 10 to 60% and necessitating annual reformulation. Conventional egg-based vaccine production is time-consuming and prone to mutations that reduce efficacy, as observed during the 2016–2017 H3N2 season when the loss of an HA glycosylation site diminished protection [112]. In contrast, mRNA vaccines offer rapid design, flexible modification, and multivalent antigen expression. Nucleoside-modified mRNA vaccines developed by Pardi et al. induced strong antibody responses targeting the HA stem domain in mice, rabbits, and ferrets. Moderna’s mRNA-1010 (50–200 µg) encodes HA proteins from A/H1N1, A/H3N2, B/Victoria, and B/Yamagata strains, producing hemagglutination inhibition titers superior to traditional inactivated influenza vaccines for influenza A and comparable for influenza B [113]. To address coinfection risks from influenza and SARS-CoV-2, Ye et al. designed a combined vaccine (AR-CoV/IAV) offering protection against H1N1 and SARS-CoV-2 α/δ variants [114]. Moderna also developed the mRNA-1073 bivalent vaccine (NCT05375838) to assess safety and immunogenicity [115]. Preclinical studies demonstrated that a single 30 µg dose of LNP-encapsulated full-length HA mRNA induced long-lasting protection against H1N1, H3N2, and H5N1 in mice [116]. mRNA-LNP influenza vaccines have thus emerged as one of the most promising clinical directions following COVID-19 mRNA vaccines [117].

#### Rabies

Rabies is an acute zoonotic disease with nearly 100% fatality. Traditional inactivated or live-attenuated vaccines require five doses, making them costly and time-intensive. Using the RNActive platform, CureVac developed the first mRNA rabies vaccine candidate, CV7201 (NCT02241135), which encodes the rabies virus (RABV) glycoprotein and uses protamine as the delivery system [118]. In a phase I trial, needle injection of 80–640 µg doses failed to induce sufficient antibody responses, whereas needle-free administration elicited strong immune responses with good safety. The second-generation vaccine CV7202 (NCT03713086) was tested in 55 healthy adults who received 1, 2, or 5 µg doses intramuscularly on days 1 and 29. Both 1 µg and 2 µg doses successfully induced neutralizing antibody responses meeting WHO standards, with good tolerability, while the 5 µg dose showed reduced reactogenicity [119]. In another study, Bai et al. developed a nucleoside-modified mRNA-LNP vaccine (RABV-G mRNA LNP) that, with a single moderate dose, elicited robust humoral and T-cell responses in mice, providing protection against lethal viral challenge and sustaining immunity for at least 25 weeks. Collectively, mRNA technology offers a promising pathway for the development of efficient, low-dose, and rapidly acting rabies vaccines [120].

#### HIV

The development of an HIV vaccine remains a formidable challenge. Despite testing more than 200 candidates over the past 40 years, none have been approved, largely due to the extraordinary antigenic diversity of the HIV envelope protein, glycan shielding of key epitopes, and the virus’s ability to integrate into host DNA [121]. The Thai RV144 trial in 2009 achieved a modest 30% protection rate but was limited by its complexity and insufficient immune potency. Recent advances in mRNA technology have renewed optimism in HIV vaccine research. Moderna has initiated several phase I trials, including eOD-GT8 60mer (mRNA-1644) and Core-g28v2 (mRNA-1644v2-Core) (NCT05001373), designed to prime B-cell maturation for broadly neutralizing antibody (bnAb) induction, as well as mRNA-1574 (NCT05217641), which encodes a native-like trimeric HIV Env. Results are pending [122]. Preclinical studies show that an mRNA-LNP vaccine encoding the modified HIV-1 Env 1086 C markedly enhances serum neutralizing activity in rabbits and macaques. Another mRNA vaccine coexpressing membrane-anchored Env and SIV Gag to generate virus-like particles (VLPs) reduced infection risk in macaques by 79% [123]. Additionally, NanoVac formulations achieved lower in vivo viral loads than conventional LNP systems. The iHIVARNA-01 vaccine demonstrated good safety and tolerability while inducing moderate HIV-specific T-cell responses, suggesting potential as an adjunct or alternative to combination antiretroviral therapy (cART) [124]. Recent studies using mRNA-LNP platforms have also successfully induced germline precursor B cells of the V3 glycan-targeting bnAb BG18, providing a new strategy for germline-targeted HIV immunization [125]. Although clinical data remain forthcoming, the unique advantages of mRNA vaccines, such as efficient antigen expression, rapid design, and the ability to elicit broad immune responses, position them as a promising breakthrough direction in overcoming the long-standing barriers to HIV vaccine development.

## Key challenges and optimization strategies

### Stability and the cold chain transportation

mRNA vaccines have achieved remarkable success in preventing severe diseases caused by SARS-CoV-2 infection, making mRNA-based drugs widely studied as potential therapeutics [126, 127]. Compared to traditional vaccines, mRNA vaccines offer several advantages such as flexible design, short development timelines, and standardized production processes [128]. However, the development of mRNA vaccines currently faces challenges, including mRNA degradation [129], strong dependence on cold chain storage, and insufficient long-term stability [130]. mRNA-LNP formulations must be stored and transported in ultra-low-temperature environments [131], such as the Pfizer/BioNTech vaccine, which requires storage at temperatures between −90 °C and −60 °C [132], and the Moderna vaccine, which requires temperatures between −50 °C and −15 °C [133]. Despite these stringent storage conditions, the shelf life of Pfizer/BioNTech and Moderna vaccines remains limited to 10–12 months [134], indicating persistent challenges in maintaining vaccine stability. This cold chain dependence severely restricts the application of these vaccines in low-resource settings and developing countries [135, 136], hindering effective immunization among low-income populations. Therefore, stability is a major bottleneck in the development and application of mRNA vaccines [137].

Vaccine stability refers to the ability of a vaccine to maintain its physical, chemical, biological, and microbiological properties over time [138]. The stability of mRNA vaccines has been investigated in both in vivo and in vitro studies. In vivo stability can be enhanced by optimizing mRNA regulatory regions (e.g., 5' cap, poly A tail, and untranslated regions [UTR]) [139], while in vitro stability is linked to key quality attributes [140], such as mRNA integrity, LNP size, charge, encapsulation efficiency, and lipid composition [141]. The stability of mRNA vaccines is influenced by multiple factors, including the molecular configuration, LNP formulation, and production process parameters [142]. The structure of mRNA affects its stability in vivo, and studies have shown that mRNA sequence characteristics (e.g., 5' cap modifications and UTR regions) significantly affect its resistance to degradation [143]. In vitro stability is closely tied to the encapsulation and delivery system, and the physicochemical properties of the LNP carrier (e.g., phase transition temperature and surface charge) directly influence the long-term storage and transport stability.

In addition to technological developments and clinical application challenges, the stability of mRNA vaccines has become a key regulatory compliance issue globally [144]. For example, the FDA guidelines for Investigational New Drug Applications require that both the drug substance and formulation maintain stable physicochemical properties during clinical trials [145] and provide verifiable degradation models. Recently, international regulatory cooperation has become more prominent, with the WHO strengthening heat stability standards for vaccines through “TRS 1011 Annex 2,” and the ICH Q5C guidelines advocating for stability risk assessment systems based on Quality by Design (QbD), such as using Monte Carlo simulations to predict the effects of storage condition changes on vaccine potency [146]. Understanding mRNA degradation pathways (e.g., 5' cap hydrolysis and lipid oxidation-induced LNP rupture) not only helps optimize current processes, but also provides mechanisms for the development of next-generation saRNA vaccines. The WHO’s updated 2022 “Evaluation of the quality, safety and efficacy of messenger RNA vaccines for the prevention of infectious diseases: regulatory considerations” introduced the concept of “dynamic stability,” using multivariate regression models (e.g., PLS-DA) to correlate environmental stress factors (e.g., temperature, humidity, and vibration) with vaccine potency decay rates [147] From a technical development perspective, analyzing phase separation mechanisms in mRNA-LNP complexes during long-term storage (e.g., lipid crystallization causing reduced encapsulation efficiency) could lead to the development of new cryoprotectants [148], thus driving the development of more durable vaccine formulations. In conclusion, stability remains the primary challenge limiting the practical application of mRNA drugs [144], and directly affect both vaccine efficacy and ongoing technological innovation [149].

#### Factors affecting the stability of mRNA vaccines

The stability of mRNA drugs results from interactions between intrinsic factors and external environmental conditions (Fig. 2). From an intrinsic perspective, the characteristics of mRNA and its delivery system, LNPs, are critical in determining stability [150]. Specifically, the chemical structure of mRNA (such as the use of modified nucleotides), the 5' cap structure, the 5' UTR, the 3' UTR, and the base sequence of the ORF significantly affect its stability. In addition, the presence of a poly(A) tail is crucial. The integrity and fragment length of mRNA, as well as the physicochemical properties and formulation of LNPs, also play important roles in their inherent stability [151]. External environmental factors primarily include storage conditions (e.g., temperature, oxidation, light exposure) and potential issues encountered during transportation (e.g., repeated freeze–thaw cycles and mechanical stress) [152]. Since LNPs are the primary delivery systems for mRNA-based drugs, this review focuses on the impact of LNP carriers and excipient components on stability.

The stability of the mRNA-LNP delivery system is influenced by chemical degradation processes (including oxidation, hydrolysis, denaturation, and deamidation) [153], and degradation mechanisms are primarily affected by pH changes, temperature, enzymes, and interactions with excipients [154]. In addition, LNPs may aggregate when stored in a solution for extended periods [155]. The factors affecting the stability of LNPs include both chemical and physical aspects. Chemical instability includes oxidation and hydrolysis of lipids in LNPs, leading to lipid degradation [156]. Lipid oxidation can occur in unsaturated fatty acid portions, potentially caused by hydrogen peroxide attacks. Oxidative impurities may also lead to the oxidation of the encapsulated mRNA. Ester bonds in lipids are prone to temperature- and pH-dependent hydrolysis. Another key aspect of LNP stability is physical degradation. The three primary types of physical instability are aggregation, fusion, and leakage of the encapsulated drug components [157]. Aggregation can arise from pH shifts and oxidation of surface lipids; controlling particle size through buffer optimization and adjustment of surface lipid density helps to minimize this risk. Leakage is mitigated by ensuring robust lipid membranes, and cryoprotectants prevent destabilization during freeze-drying and long-term storage. Aggregation of LNPs has been reported during storage [158]. To enhance their stability, LNPs are typically formulated with PEG-lipids to improve colloidal stability and reduce particle aggregation [159]. Another type of physical instability is mRNA leakage, which can compromise the integrity of the encapsulated product. Encapsulation efficiency (EE) is an important quality attribute of mRNA-LNP formulations and is influenced by formulation, mixing, and processing parameters [160]. During long-term storage, leakage of encapsulated mRNA can increase the fraction of unencapsulated mRNA and consequently decrease EE over time [161].

##### Impact of LNP components

**Fig. 3:**
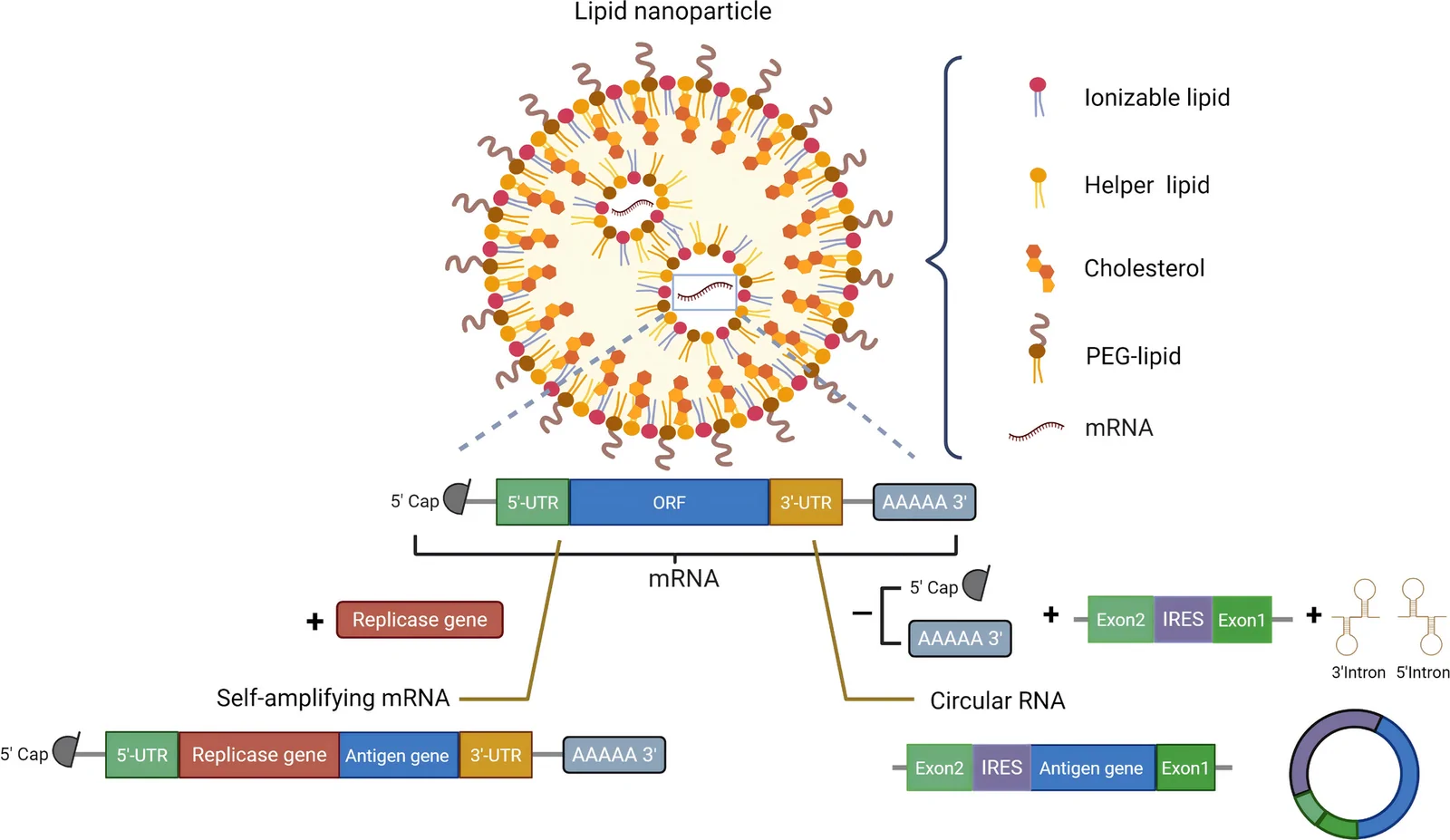
Schematic illustration of lipid nanoparticle structure and in vitro transcribed (IVT) mRNA components. LNPs are composed of four classic constituents: ionizable lipids, cholesterol, PEG lipids, and helper lipids. The IVT mRNA is characterized by its open reading frame (ORF), 5' and 3' untranslated regions (UTRs), a 5' cap structure, and a 3' poly(A) tail. Each element of the mRNA can be precisely engineered to enhance translation efficiency, stability, and modulate immunogenicity

The stability of mRNA-LNPs is governed by the physicochemical properties of their four classic components: ionizable lipids, cholesterol, PEG lipids, and helper lipids [162] (Fig. 3). These components influence formulation stability through their effects on lipid membrane organization, chemical degradation pathways, and interactions with encapsulated mRNA.

Ionizable lipids are the core functional components of mRNA-LNPs. Chemical instability primarily arises from several mechanisms: Ionizable lipids are essential for mRNA encapsulation and intracellular delivery; however, their chemical structures also influence LNP stability. Ester/amide bonds within lipid structures may undergo hydrolysis, while unsaturated hydrocarbon chains are susceptible to oxidative degradation under thermal or oxidative stress [163]. These degradation processes may alter lipid integrity and subsequently affect the stability of the overall mRNA-LNP formulation [164].

Cholesterol contributes to LNP structural stability by regulating membrane packing, fluidity, and mechanical integrity [165]. Appropriate cholesterol content helps maintain particle stability, whereas an imbalance in cholesterol composition may affect membrane organization and formulation performance [166, 167]. In addition, excess cholesterol or cholesterol derivatives have been reported to promote cholesterol-rich crystalline domains or crystallite formation within LNPs, which may alter membrane organization and affect particle stability [168, 169].

PEG-lipids confer steric stabilization that reduces particle aggregation during formulation, storage and handling, and can modulate in vivo transport and transient systemic exposure after administration [170]. A notable drawback is that the polyethylene oxide (PEO) ether bonds in PEG chains are susceptible to oxidative cleavage; free-radical attack can generate small reactive carbonyl species (e.g., acetaldehyde, formic acid) that react with nucleic acids or buffer components [171]. Such reactions have been reported to form adducts with nucleobases (which may impair mRNA function) and Schiff-type products with buffer amines (which can affect buffer pH stability), thereby compromising mRNA integrity and overall formulation stability [172].

Phospholipids are crucial for enhancing the stability of the lipid bilayer and improving the nucleic acid encapsulation efficiency. The chemical instability of these phospholipids poses a challenge for long-term storage of the formulation: (1) unsaturated olefin chains are susceptible to free radical-mediated chain oxidation, producing α,β-unsaturated aldehydes (such as 4-hydroxy-2-nonenal) and short-chain carboxylic acid derivatives [173]. These electrophilic products can undergo Michael addition reactions with mRNA bases (such as the primary amine of guanine), leading to nucleic acid crosslinking; (2) During formulation production or storage, if the system pH fluctuates significantly (e.g., local pH < 5 or > 9), the protonation state of the phospholipid head group may change, causing a sharp increase in electrostatic repulsion between lipid molecules. This, combined with thermal stress (e.g., repeated freeze–thaw cycles), can cause hydrolysis of the glycerol backbone ester bonds, releasing free fatty acids (e.g., oleic acid) [174]. The latter not only disrupts the LNP structure through micellization, but may also activate intracellular inflammatory pathways, increasing the immunogenic risk of the delivery system.

##### Impact of formulation process

The influence of the encapsulation process on LNP stability primarily involves the following factors: LNP particle size, surface charge, type of buffer solution, pH, ionic strength, and residual amounts of ethanol and citric acid in the preparation process [175].

First, the particle size of LNPs is a critical factor affecting their physical stability. Encapsulation methods, such as microfluidic mixing and ethanol injection, affect the particle size and dispersion of LNPs [176]. Overly large or uneven particles are prone to aggregation or sedimentation, leading to physical instability. Larger particles are more likely to settle under the influence of gravity, especially when left standing for extended periods. The ideal particle size range for LNPs is typically controlled between 50–150 nm (e.g., 80–100 nm for Moderna/Pfizer vaccines), and uniformity can generally be achieved by optimizing parameters such as flow rate and mixing ratio. If the particle size exceeds this range, aggregation and sedimentation are more likely to occur during storage [177].

Second, the surface charge of LNPs is also a significant factor influencing their physical stability.Surface charge regulates aggregation and cellular uptake; modulating lipid composition to optimize charge enhances mRNA-LNP delivery. The surface charges of the LNPs changed under different pH conditions.The surface charge of LNPs is pH-dependent; under acidic conditions, protonation of ionizable lipids increases positive surface charge and enhances electrostatic repulsion [178]. Similarly, an excessively high proportion of cationic lipids may disturb the physicochemical balance of the LNP formulation, alter surface properties and interparticle interactions, and thereby increase the risk of aggregation and sedimentation. Adjusting the ratio of PEG lipids can stabilize the surface charge and reduce nonspecific adsorption [179]. High-ionic-strength environments can screen the electrostatic repulsion between LNP particles, leading to aggregation and sedimentation.

During the encapsulation process, LNPs are primarily prepared using microfluidic or shock-flow technology. Nucleic acids are typically dissolved in a citric acid solution, whereas lipids are dissolved in an ethanol solution. The two solutions were then mixed using these techniques to form mRNA-LNPs, followed by dialysis, ultrafiltration, or tangential flow filtration (TFF) to remove most of the ethanol and citric acid. However, residual ethanol and citric acid may remain [180]. Generally, the ethanol content of the final product should be less than 0.5% (v/v). Higher ethanol concentrations can lead to pharmacopoeia compliance issues (e.g., ICH Q3C standards) and negatively affect the long-term stability of LNP formulations. Ethanol can also cause lipid membrane fusion in LNPs, disrupting the hydrophobic regions of the lipid bilayer, leading to loosening or rupture of the LNP structure, resulting in an increase in particle size (aggregation) or leakage of encapsulated mRNA. It may also cause lipid oxidation and hydrolysis, which can degrade the mRNA.Residual citric acid buffer may create localized acidic conditions that promote lipid hydrolysis and mRNA degradation, potentially compromising LNP stability [181].

##### Storage and transportation

The stability of mRNA-LNP formulations during storage and transportation is primarily affected by temperature, light exposure, and mechanical stress, with an evident impact on the stability differences observed in the marketed vaccines. Data from the Moderna mRNA-1273 and Pfizer BNT162b2 vaccines revealed significant temperature sensitivity: the former maintains structural integrity at −20 °C for six months, but stability drops sharply to 30 days at 2–8 °C; the latter requires ultra-low-temperature storage (−80 °C to −60 °C), with a shelf life of only five days under standard refrigeration conditions (2–8 °C) [182]. Research teams have experimentally assessed the impact of temperature ranges from −20 °C to 40 °C on mRNA vaccine stability. The results showed mRNA degradation in all samples, with higher temperatures leading to a significant decline in integrity. Notably, a similar nucleic acid drug, Onpattro siRNA, maintains efficacy for up to three years under 2–8 °C storage conditions [183], suggesting that the intrinsic structural characteristics of mRNA molecules may be a key factor limiting their stability. To address this issue, Zhang et al. [184] employed LNP encapsulation technology to develop a novel mRNA vaccine formulation encoding the SARS-CoV-2 RBD domain. This formulation maintained stability for seven days at room temperature and retained immunogenicity even after one month of refrigeration. Further experiments showed that when stored for up to six months (2–8 °C), key parameters, such as mRNA purity, encapsulation efficiency, and polydispersity index (PDI), remained stable, and antibody responses in animal models remained consistent. This temperature dependence results from two main degradation pathways: on the one hand, elevated environmental temperatures accelerate free radical-mediated chain oxidation of unsaturated components in the lipid bilayer, producing aldehyde derivatives (such as malondialdehyde) that induce covalent modification of mRNA bases [185]; on the other hand, increased temperatures disrupt nucleic acid secondary structures (such as the protective topology of the 5' cap and poly(A) tail), increasing the potential for ribonuclease attack on vulnerable sites [186].

In addition to temperature factors, mechanical stress during transportation (such as vibration or impact) may adversely affect the physical stability of the LNPs,vibration-induced aggregation can compromise LNP stability; proper storage conditions and buffer optimization are crucial for mitigating this effect. Studies have shown that LNPs are prone to aggregation or rupture during transportation, which leads to decreased bioactivity. Mechanical stress during transport (such as 180 min of vibration or severe shaking) weakens colloidal electrostatic repulsion, promoting particle aggregation, resulting in a broader particle size distribution (PDI > 0.25) and decreased mRNA encapsulation efficiency (with free RNA content increasing to over 15%) [187]. Kudsiova et al. [188] further confirmed that repetitive syringe operations could induce lipid membrane fusion, causing LNP structural breakdown and mRNA chain fragmentation. Furthermore, freeze–thaw cycles subject the lipid bilayer to mechanical stress from ice crystal growth, compromising lipid bilayer continuity and significantly altering physicochemical properties (such as zeta potential and particle size). Therefore, maintaining a low-temperature, light-protected environment and avoiding physical shocks (such as vibration, shear, and freeze–thaw cycles) is an essential strategy for ensuring the stability of mRNA-LNP formulations from production to clinical application, shear and impact forces during transport can destabilize LNPs, causing aggregation or leakage; optimizing lipid composition minimizes these effect.

##### ***Impact of LNP manufacturing technologies on stability***

The formulation technology of LNPs plays a pivotal role in determining their physicochemical properties, encapsulation efficiency, and stability, thereby directly influencing LNP quality and performance. The principal formulation techniques employed in both laboratory and industrial settings include pipette mixing, vortex mixing, thin-film hydration, ethanol injection, microfluidics, and impingement jet mixing [189].

Focusing on strategies commonly used in research and manufacturing scenarios: pipette and vortex mixing allow for rapid benchtop-scale preparation with simple procedures and minimal equipment requirements. However, the resulting high polydispersity and batch-to-batch variability constrain scalability and encapsulation efficiency. The thin-film hydration process is a well-established method characterized by low residual solvent levels, yet its scalability is limited by the time-consuming solvent evaporation and homogenization steps [190]. Ethanol injection initiates self-assembly via rapid dilution, with particle size governed by lipid concentration, stirring or injection rate, and formulation composition. Nevertheless, traditional ethanol injection methods suffer from limited reproducibility and scalability [191].

Impingement jet mixing (IJM) achieves instantaneous mixing and scalable continuous production through the collision of opposing high-speed jets and has been validated in mRNA vaccine manufacturing. The microfluidic mixing technique offers precise control over the mixing process, enabling uniform LNP size distribution and minimizing batch-to-batch variation [192]. However, microfluidic devices are typically expensive and require substantial initial capital investment. In addition, channel clogging(often caused by lipid aggregation)can necessitate further maintenance expenditures. Although the use of disposable microfluidic chips can mitigate clogging issues, these components are costly. Furthermore, syringe-based microfluidic systems may exhibit dead volumes, resulting in material waste [193].

By utilizing microfluidic pathways such as hydrodynamic flow focusing, staggered herringbone, and T-junction mixing, researchers achieve controlled microscale convection/diffusion and enhanced turbulence. This allows fine programmability of particle size and key attributes, while boosting consistency and throughput [194]. Overall, the spectrum of techniques from laboratory to industrial-scale production reflects an evolution “from simplicity to precision.” Achieving a balance between speed, convenience, reproducibility, scalability, and high encapsulation efficiency remains essential. Continuous manufacturing platforms such as microfluidics and IJM offer superior solutions to meet the dual demands of product quality and production capacity. A summary of preparation methods and their respective advantages and disadvantages is provided in Table 3.

**Table 3:** Analytical techniques for mRNA-LNP stability assessment

Preparation method	LNP application	Operation	Advantages	Disadvantages	References
Pipette Mixing	Rapid formulation technique; suitable for small-batch laboratory preparation	Mix a lipid-containing organic phase (ethanol) with a nucleic-acid buffer and rapidly agitate by pipetting to homogenize	Simple operation, short processing time, and no expensive equipment required	Not suitable for large-scale manufacturing	[189]
Vortex	Rapid formulation technique; suitable for small-batch laboratory preparation	Combine the nucleic-acid buffer with the lipid–ethanol solution and vortex at a moderate speed to homogenize	Easy to implement and does not require specialized instrumentation	Not suitable for large-scale manufacturing	[189]
Thin-Film hydration	Classical technique for preparing lipid nanoparticles	Dissolve lipids in an organic solvent and evaporate the solvent to form a thin lipid film on the vessel wall; then hydrate the film with a nucleic-acid aqueous solution, during which the swollen film curls and closes into vesicular structures; finally, apply extrusion or sonication to obtain uniformly sized LNPs	Low solvent residue and cost-effective	Solvent evaporation is time-consuming (several hours), limiting scalability	[190]
Ethanol injection	General method for LNP preparation with broad applicability	Dissolve lipids in ethanol to prepare the lipid phase and, under continuous stirring, rapidly inject it into an aqueous buffer containing nucleic acids to achieve instantaneous encapsulation; subsequent rapid dilution in the aqueous phase increases polarity, driving spontaneous self-assembly of lipids into vesicles	Operationally simple and does not require costly equipment	Manual injection yields poor batch-to-batch reproducibility, unsuitable for industrial production	[191]
Hydrodynamic flow focusing(HFF)	Microfluidic technique for LNP preparation	Operate on a capillary microfluidic platform in which a central lipid stream is hydrodynamically focused by flanking aqueous streams containing nucleic acids, enabling spontaneous LNP formation; particle size can be precisely tuned by adjusting the flow-rate ratio and total flow rate	No need for post-processing such as sonication, extrusion, or filtration; suitable for industrial production with high batch-to-batch consistency	Instrument cost is high and may be incompatible with very large-scale manufacturing	[194]
Staggered herringbone micromixers (SHM)	Microfluidic technique enabling high-throughput LNP production	Inject the lipid solution and the nucleic-acid aqueous buffer into a microchannel patterned with asymmetric staggered herringbone grooves on the channel walls, which induce chaotic advection for efficient mixing and yield LNPs with uniform size	Good reproducibility; suitable for manufacturing	High instrument cost	-
T-Junction mixing	Microfluidic technique suitable for LNP production	Introduce the lipid solution and the nucleic-acid aqueous buffer through two independent microchannels at high velocity; the two streams impinge at the T-junction, generating intense collisions that result in instantaneous mixing and reproducible LNP formation			[193]
Impingement jet mixer (IJM)	Innovative continuous-flow mixing approach; applicable to scalable LNP manufacturing	Mix the lipid phase with the aqueous phase and drive them through opposing high-pressure jets to generate fine droplets that form LNPs; physicochemical properties of the nanoparticles can be tuned by adjusting mixer parameters			[192]

#### Optimization of key components of LNP stability

##### Ionizable lipids

To maintain the long-term stability (including both chemical stability and biological activity) of the mRNA/LNP system, it is essential to overcome the intrinsic instability of mRNA and prevent unintended reactions between the lipid impurities and mRNA. Ionizable lipids with tertiary amines generate aldehyde impurities via oxidation and hydrolysis. These aldehyde impurities can covalently bond with mRNA, damaging its activity and integrity during storage [195]. This mechanism accelerated mRNA degradation and decreased the stability of the mRNA/LNP system. The amine groups in ionizable lipids are responsible for the production of aldehydes [196]. Therefore, designing amine structures to limit the oxidation and hydrolysis of ionizable lipids and the resulting aldehyde production can enhance the long-term stability of mRNA/LNP systems at high temperatures.

Research has shown that the formation of "bleb" structures in LNPs can be intentionally induced to enhance stability and potency. Cheng et al. [197] demonstrated that formulating LNPs in high-concentration pH 4 buffers (e.g., 300 mM sodium citrate) can induce bleb structures which encapsulate mRNA, leading to significantly improved mRNA integrity and transfection efficacy both in vitro and in vivo. They proposed that this is due to a phase-separation mechanism that sequesters mRNA into the bleb's aqueous core, thereby shielding it from degradation.However, the formation and functional impact of blebs are highly context-dependent, and not all blebs are equivalent. As highlighted by Simonsen [198] the propensity to form blebs varies considerably among ionizable lipids; for instance, ALC-0315 readily forms blebs under standard buffer conditions, whereas SM-102 requires high ionic strength to do so significantly. Crucially, the literature presents a nuanced picture: while mRNA-loaded blebs formed under optimized conditions (as in Cheng's work) are associated with enhanced stability and potency, blebs induced by stress conditions (e.g., freeze–thaw) are often empty and correlate with reduced performance. Therefore, the positive attributes of blebs are contingent upon the specific formulation parameters that ensure both their formation and successful mRNA loading. This underscores that optimizing ionizable lipids and formulation processes to reliably generate mRNA-loaded blebs could be a valuable strategy for enhancing LNP stability (Fig. 2).

Lipids with branched alkyl chains enhance the physical stability of LNPs by reducing the mobility of lipid molecules, thereby strengthening the overall particle structure [199]. As mentioned earlier, ionizable lipids consist of three parts: a hydrophilic head containing nitrogen-based groups, a hydrophobic tail composed of fatty acid chains, and a linker [200]. Branched lipids increase the stability of LNPs through several mechanisms [201]. First, the branched structure increases the microviscosity of the LNPs. This increased viscosity restricts lipid movement, thus slowing the degradation of LNPs in the body. Specifically, the microviscosity of the mRNA-LNP system has a significant positive correlation with formulation stability. When the microviscosity exceeded 150 mPa · s, the particle size, polydispersity index (PDI), and mRNA encapsulation efficiency (EE) remained stable after 30 d of storage at 40 °C. However, when the microviscosity fell below this threshold, the particle size and PDI increased significantly and the EE decreased. The complex structure of the branched tails led to denser molecular packing, making the LNP more compact and stable. Mechanistic analysis shows that, compared to linear tail chains, branched tails effectively limit the rotational freedom of lipid molecules within the lipid bilayer, creating a higher-viscosity microenvironment inside the LNPs [202]. This mechanism may explain the superior stability of the mRNA-LNP systems containing branched-tail ionizable lipids.

Impurities in ionizable lipids (such as aldehydes) may react with mRNA, leading to the inactivation of nucleic acids and instability of LNPs. By optimizing the synthesis pathway or introducing protective groups (such as piperidine groups), the formation of these impurities can be reduced, thereby enhancing the stability of LNPs [203]. The amine groups in the ionizable lipids limit the generation of aldehyde impurities. As these aldehyde impurities typically react with mRNA nucleotides during storage, causing mRNA functional loss and reducing mRNA-LNP stability, this has been confirmed by high-performance liquid chromatography (HPLC) analysis. Lipids with piperidine structures can effectively reduce the formation of aldehydes, thereby decreasing the likelihood of reactions with mRNA. Experimental results showed that at refrigerated temperatures (for liquid formulations), piperidine-based lipids significantly improved the long-term storage stability of mRNA/LNP systems. This stability enhancement was primarily attributed to the effective inhibition of aldehyde impurity formation by the amine group, which reduced mRNA degradation and functional loss during storage.

##### Cholesterol

As a key constituent of mRNA-LNPs, cholesterol plays a dual role in regulating particle stability and enhancing mRNA expression efficiency. Experimental data indicated that when the cholesterol concentration fell below 10 mol%, the physical stability of mRNA-LNPs significantly decreased, leading to larger particle sizes and reduced mRNA encapsulation efficiency. Notably, excessively high cholesterol concentrations can weaken the stability of lipid membranes, rather than enhance their performance. Therefore, optimizing the mRNA-LNP formulation requires balancing cholesterol content to avoid structural instability caused by either too little or too much cholesterol [204].

Berti et al. [205] developed two LNP formulations, in which 25% of cholesterol was replaced by the anti-inflammatory drug dexamethasone (DX). Cholesterol typically plays a role in stabilizing the structure of LNPs by regulating membrane fluidity and preventing lipid fusion, thereby enhancing particle stability. Although part of the cholesterol was replaced by DX, DX, as a steroid molecule, may retain cholesterol-like functionality. The experimental results showed that the prepared LNPs exhibited near-neutral zeta potentials. This charge characteristic helps to prevent electrostatic repulsion or attraction between particles, avoiding excessive electrostatic forces that could lead to aggregation or dissociation, thereby maintaining particle uniformity and dispersion. SAXS experiments confirmed that DX was successfully incorporated into the LNP core to form a stable internal structure. The slow-release behavior of DX indicated a uniform and stable distribution within the LNP, reducing the risk of structural disruption due to drug leakage. Further experiments using DLS measurements showed that the LNPs displayed an excellent particle size distribution and homogeneity. Smaller and more uniform particles not only favor cellular uptake, but also reduce interactions between particles, further enhancing the overall stability. Consequently, the resulting LNPs demonstrated high stability, homogeneity, and near-neutral zeta potential, achieving 95–100% high mRNA encapsulation efficiency, making them more suitable for in vivo delivery.

Jung et al. [206] developed a new ionizable cholesterol analog(3β[L-histidine amide-carbamoyl] cholesterol,Hchol) and incorporated it into LNPs as a replacement for traditional cholesterol. This design significantly improved the functional stability and delivery efficiency of LNPs. Hchol was introduced into cholesterol via a biodegradable carbamate linkage incorporating a histidine amine group. Hchol can replace cholesterol in SM102 mRNA-LNPs to form SM-Hchol or can be used in MC3 mRNA-LNP formulations to form MC3-Hchol. The stabilization mechanism of Hchol mainly works through several aspects. First, the pKa value of Hchol is optimized between 6.03 and 6.61, making it better protonated in the acidic environment of endosomes (usually pH around 5–6). This protonation enhances the interaction between the lipid particles and the endosomal membrane, facilitating endosomal escape and improving the overall stability. Second, Hchol increased endosomal escape efficiency. The histidine amide group in Hchol contains an imidazole ring that protonates under acidic conditions. The protonated imidazole group attracts anions, leading to the so-called “proton sponge effect,” which disrupts the stability of the endosomal membrane, allowing mRNA to escape more effectively from the endosome into the cytoplasm. Increased endosomal escape efficiency directly reduces the risk of mRNA degradation in the endosome, enhancing the overall stability of the delivery system. Additionally, the synergy between the protonated imidazole group of Hchol and cholesterol further strengthens its ability to disrupt the endosomal membrane. This synergistic effect not only improved the permeability of the endosomal membrane, but also enhanced the overall structural stability of the LNPs. The experimental results showed that Hchol-LNPs exhibited a stronger red blood cell lysis ability at pH 5.5, indicating a significantly enhanced ability to disrupt the endosomal membrane. In mouse models, mRNA delivered via Hchol-LNPs showed significantly improved delivery efficiency, suggesting that Hchol enhanced LNP stability in in vitro experiments.

##### Buffer solutions

Selecting the appropriate buffer system and osmolality adjusters is critical in mRNA-LNP formulations, as mRNA vaccines typically require storage at low temperatures, and freezing can cause significant pH shifts. Initially, Pfizer's COVID-19 vaccine used a phosphate-buffered saline (PBS) system containing KCl/NaCl. However, due to significant pH fluctuations observed after freezing with phosphate buffer, a switch to Tris buffer was made [207]. Tris–HCl not only stabilizes nucleic acids but also has the ability to scavenge hydroxyl free radicals, which further enhances the stability of the formulation [208].

Buffer solutions significantly affect the stability of mRNA/LNP formulations by regulating pH, reducing aggregation, protecting membrane integrity, and enhancing freeze–thaw and long-term storage stability. Selecting an appropriate buffer solution is one of the key steps in optimizing the performance of the LNPs [209]. Tang et al. [210] used dynamic light scattering (DLS) to measure the particle size distribution and aggregation of LNPs under different buffer conditions. They found that in inappropriate buffer solutions (such as low pH or high ionic strength), LNPs showed noticeable aggregation with a significant increase in particle size. In contrast, when optimized buffer solutions were used, LNPs maintained good dispersion and stable particle size. By encapsulating fluorescence-labeled mRNA into LNPs, they performed freeze–thaw cycles or long-term storage tests and monitored mRNA leakage. The results showed that certain buffer solutions caused the LNP membrane structure to break down, increasing mRNA leakage. In contrast, when optimized buffer solutions were used, the LNPs maintained good dispersion and stable particle size. Moreover, Tris-based buffers were shown to enhance the freeze–thaw stability of LNPs, significantly reducing particle size changes and mRNA leakage during freeze–thaw cycles.

The stability and performance of the mRNA-LNP formulations were significantly affected by the buffer solution. Research has shown that the components of buffer solutions may cause lipid-water phase separation during freeze–thaw cycles, thus affecting the storage stability. To assess the effect of buffer solutions on the performance of mRNA-LNP formulations, Henderson et al. [211] prepared formulations using PBS, TBS, and HBS buffers. The results showed that the initial particle size (approximately 70 nm), encapsulation efficiency (> 92%), and morphology were consistent across all three buffer solutions, with a PDI of 0.05–0.10. Surface charge analysis revealed that the PBS and TBS formulations were slightly negatively charged, whereas the HBS formulation was neutral. After storing at −20 °C for 3 weeks and thawing, the TBS formulation maintained a stable particle size, while the PBS and HBS formulations increased by 50%. Cryo-transmission electron microscopy revealed that the PBS formulation exhibited an uneven internal structure with lipid-water phase separation, whereas the TBS formulation displayed fused nanoparticle structures, and the HBS formulation formed an "hourglass" structure. Optimization of the Pfizer/BioNTech vaccine from PBS to TBS addressed the freezing instability issue and significantly extended the shelf life. Additionally, Tris in TBS captured aldehyde impurities, reducing the formation of mRNA-lipid adducts and enhancing their stability. This study underscores the importance of buffer solution selection in optimizing the freeze-storage stability of mRNA-LNP formulations.

##### Stabilizing agents

The selection and optimization of stabilizing agents are crucial for ensuring the long-term stability of mRNA-LNP formulations, especially in the storage and transportation processes. Research indicates that sugars are the most commonly used stabilizing agents and exhibit significant cryoprotective effects during freeze–thaw cycles and lyophilization processes [212]. For example, Moderna and Pfizer/BioNTech COVID-19 vaccines use 8% and 20% w/v sucrose as stabilizing components, respectively. Currently, there are three main theoretical models explaining the protective mechanisms of sugar compounds: the vitrification theory, the particle separation hypothesis, and the water molecule replacement mechanism. Vitrification theory suggests that sugars can form a glass-like matrix on the surface of nanoparticles, effectively preventing particle aggregation and inhibiting ice crystal formation.Vitrification during freeze-drying, aided by stabilizers like sucrose, prevents aggregation and enhances the structural integrity of LNPs. The water molecule replacement mechanism proposes that sugars interact with the polar head groups of lipid molecules, thus maintaining the structural stability of nanoparticles.According to the particle separation hypothesis, sugars become concentrated in the unfrozen phase during freezing and physically separate adjacent nanoparticles, thereby reducing particle aggregation or fusion [213]. The water molecule replacement mechanism proposes that sugars form hydrogen bonds with the polar head groups of phospholipids, partially replacing water molecules during dehydration and thereby maintaining membrane structural stability [214].

Kafetzis et al. [215] found that using stabilizing Agents can effectively prevent nanoparticles from losing functionality or degrading under various storage conditions. Adding sucrose can keep all types of nanoparticles stable at −80 °C for up to one month, maintaining their therapeutic efficacy irrespective of whether they carry DNA or mRNA. Sucrose plays a critical role as a cryoprotectant for maintaining the structural integrity, encapsulation efficiency, and functional performance of lyophilized mRNA-LNPs. The low-temperature protective ability of sugars varies according to type, with sucrose and trehalose being more effective than mannitol. For instance, 20% w/v sucrose helps lyophilized mRNA-LNPs maintain a stable appearance, whereas mannitol causes instability in the colloidal structure [216]. Furthermore, excessively high sugar concentrations (such as 40% w/v) may reduce the mRNA transfection efficiency, although the particle size remains consistent. Lyophilized mRNA-LNPs maintained similar in vitro activity as fresh formulations, but in vivo activity significantly decreased, suggesting that the lyophilization process may alter the structure and affect transfection efficiency. Non-lyophilized mRNA-LNPs stored in liquid nitrogen maintained the same transfection efficiency under both in vitro and in vivo conditions, highlighting their advantages. Studies have shown that adding 12% w/v sucrose or trehalose ensures the stability of mRNA-LNPs at −80 °C for two weeks, with no significant changes in size, surface charge, encapsulation efficiency, or transfection capability [217]. However, sucrose and trehalose did not improve the stability of liquid mRNA-LNPs at room temperature or at 4 °C. Another study confirmed that 10% w/v sucrose could maintain the activity of LNP formulations at −20 °C for at least 30 d [218]. During lyophilization, 20% w/v sucrose aided in the successful reconstitution of mRNA-LNPs, maintaining particle size and encapsulation efficiency, while the absence of sucrose led to a significant loss in encapsulation efficiency. The type of ionizable lipid also affects the encapsulation stability during mRNA lyophilization. For example, S − Ac7-Dog mRNA-LNPs maintain 100% encapsulation after lyophilization, whereas S − Ac7-DHDA mRNA-LNPs lose approximately 40% of the encapsulated mRNA [219].

Stabilizing Agents are crucial in the development of mRNA-LNP formulations to maintain long-term stability during storage and transportation. To ensure mRNA stability, all formulation components must be of high purity to avoid contamination by heavy metals, residual solvents, or endotoxins, which can affect therapeutic efficacy [220].

##### pH

Although the pH of the final LNP formulation is generally maintained within a defined buffer system during storage, precise pH control is particularly important during LNP manufacturing, especially during particle self-assembly and subsequent buffer exchange, because pH directly affects ionizable lipid protonation, mRNA encapsulation, and particle stability.Feng et al. [221] conducted atomistic molecular dynamics (MD) simulations to study the pH-dependent structural transitions and water permeability behavior of mRNA LNPs in aqueous solutions. They discovered that the ionizable lipid (IL) membrane of the LNPs is highly sensitive to pH. As the environmental acidity increased, the LNPs membrane rapidly collapsed and underwent hydration expansion. This change disrupts the structural integrity of LNPs, thereby affecting their stability. As the acidity increases, the structure of the LNPs becomes looser, which allows water molecules to penetrate more easily into the LNP core. High water permeability further decreases the stability of LNPs in non-frozen liquid environments because the presence of water may accelerate mRNA degradation. Tang et al. [210] found that when the pH is 4, the encapsulation efficiency and intracellular expression levels of LNPs are highest. This indicates that the pH regulation of the buffer solution is critical for the initial formation and functionality of LNPs. Larson et al. [204] observed that when the pH is below the pKa of cationic lipids, the particle size of mRNA-LNPs increases and aggregation is more likely to occur, further highlighting the importance of pH control in maintaining LNP colloidal stability. Collectively, these findings highlight the importance of precise pH control during LNP formulation, particularly during self-assembly and subsequent buffer exchange.

##### Ethanol

During the encapsulation process, LNPs are typically prepared using microfluidic or jet mixing technologies, with nucleic acids dissolved in citrate solution and lipids dissolved in ethanol. The resulting mixture forms mRNA-LNPs, which are then processed to remove most of the ethanol and citrate by dialysis, ultrafiltration, or tangential flow filtration (TFF). However, traces of ethanol and citrate may remain in the final product, with ethanol levels generally required to be less than 0.5% (v/v) [222]. Higher levels of ethanol could lead to issues with pharmacopoeial compliance (e.g., ICH Q3C standards) and negatively impact the long-term stability of the LNP formulation. Ethanol can also cause lipid membrane fusion, disrupting the lipid bilayer by dissolving hydrophobic components, leading to a loss of LNP structural integrity. This results in an increased particle size (aggregation) and mRNA leakage [76]. Additionally, ethanol contributes to lipid oxidation and hydrolysis, further promoting mRNA degradation. Residual citrate buffer may lower the pH of the system, reduce the surface charge of LNPs, weaken the electrostatic repulsion between particles, and increase the risk of aggregation and precipitation [223].

### Safety and reactogenicity profile

#### Clinical reactogenicity and adverse events of mRNA-LNP vaccines

Vaccine reactogenicity refers to the physical manifestations of the inflammatory response triggered by the vaccine, including both systemic and local side effects. Systemic side effects such as fatigue, headache, fever, myalgia, and arthralgia are commonly observed, while local side effects include swelling, redness, pain, and warmth at the injection site. These effects are more pronounced in younger individuals and tend to intensify after multiple doses. Although severe adverse events such as acute myocardial infarction, cerebral venous sinus thrombosis, myocarditis/pericarditis, pulmonary embolism, Guillain-Barré syndrome, stroke, and anaphylactic shock are rare, they can have significant consequences. mRNA itself plays a substantial role in mediating reactogenicity, which is primarily driven by the activation of innate immune pathways. The activation of immune sensors triggers the release of pro-inflammatory cytokines, including TNF-α, IL-6, and interferons, which are mainly responsible for symptoms such as fever, headache, and muscle pain [224]. Even FDA-approved commercial LNP carriers, such as BNT162b2, exhibit reactogenicity. Therefore, optimizing reactogenicity to balance therapeutic efficacy with clinically acceptable risk levels is crucial.

#### Mechanistic determinants and formulation strategies to improve safety

A deeper understanding of the mechanistic basis of reactogenicity is essential for guiding formulation optimization and improving the overall safety profile of mRNA-LNP vaccines. Abbasi et al. [225] addressed the critical issue of reactogenicity in conventional mRNA-LNP vaccines by using a needle-free, pyro-driven liquid jet injector (PYRO) to target skin layers enriched in antigen-presenting cells (APCs). This approach delivers naked mRNA directly into APCs, which subsequently migrate to lymph nodes to present antigen, thereby substantially reducing the risk of reactogenicity. Kawaguchi et al. [226] demonstrated that modifying the composition and structural features of PEG lipids, cholesterol, and phospholipids can markedly influence the reactogenicity of mRNA-LNP vaccines. Shortening the PEG chain length and lowering the molar ratio of PEG lipids enhanced immune responses while reducing adverse reactions. Metabolites of ionizable lipids, such as fatty acids, may induce toxicity through activation of peroxisome proliferator-activated receptors (PPARs) and can also promote hepatic neutrophil infiltration, leading to elevated alanine aminotransferase (ALT) and aspartate aminotransferase (AST), two established markers of liver injury [227]. In addition, PEGylated lipids alter the pharmacokinetics and biodistribution of LNPs. Repeated administration of PEGylated LNPs can trigger immune responses, generating anti-PEG antibodies and increasing the risk of adverse events due to unintended biodistribution [228]. Finally, the mRNA component of LNPs may cause off-target effects, innate immune activation, and excessive protein expression, contributing to toxicity. Off-target delivery leads to antigen expression in non-APCs, eliciting unintended immune responses and tissue-specific injury. Moreover, mRNA is inherently unstable and prone to degradation, releasing RNA fragments [87]. These fragments can function as damage-associated molecular patterns (DAMPs), further activating immune pathways and promoting toxicity, ultimately triggering excessive cytokine release and systemic inflammation.

Xue et al. [229] designed ionizable lipids capable of reducing toxicity in non-target tissues while improving delivery efficiency. They developed a series of structurally distinct siloxane-based ionizable lipids and formulated siloxane-incorporated LNPs to enhance the potency of LNP-based therapies while minimizing toxicity. Vitamin B5 exhibits protective effects against oxidative stress, mitochondrial toxicity, and cardiovascular injury. For this reason, vitamin B5-derived ionizable lipids may support the development of safer LNPs for mRNA vaccine delivery.Yoo et al. [226] synthesized several innovative vitamin B5-derived ionizable lipids with low toxicity as core structural components of LNPs, demonstrating favorable physicochemical properties and high mRNA transfection efficiency. Nogueira et al. [77] replaced PEGylated lipids with polysarcosine (pSar), a polymer derived from the endogenous amino acid sarcosine. This substitution reduced the risk of hypersensitivity reactions and complement activation-related pseudoallergy (CARPA). The PEG-free design improved protein expression and safety, making pSar-functionalized LNPs a promising low-toxicity platform.In summary, these findings highlight that rational lipid design and careful modulation of LNP composition are central to achieving safer mRNA-LNP platforms without compromising therapeutic efficacy.

### Durability of immune response

#### Enhancing immunogenicity and immune memory to sustain durable protection

The effectiveness of LNP-based therapies is contingent on overcoming hurdles related to physicochemical stability, delivery efficiency, and immunological compatibility. These challenges are interconnected and must be addressed to enhance the durability of the immune response. Given that physicochemical stability has been discussed previously, this section focuses on immunogenicity and immune compatibility.

Immunogenicity refers to the ability of a substance to elicit an immune response. A robust immune response is essential for vaccine effectiveness, and enhancing immunogenicity is particularly critical in vaccines intended to protect against infectious diseases that require strong immune activation, including pathogens with high virulence or mutation rates, as well as populations with weaker immune responses. Stronger immunogenicity can broaden protection across multiple pathogens or variants and prolong immunity, thereby reducing the need for frequent booster doses. Maintaining high immunogenicity also enables vaccines to remain effective in immunocompromised individuals. Li et al. [230] combined the adjuvant properties of ionizable lipids with those of mRNA itself, employing TLR agonists to directly activate dendritic cells and enhance antigen presentation, ultimately strengthening adaptive immune responses. Adjuvants create a localized immunostimulatory environment at the injection site, thereby significantly augmenting both innate and adaptive immunity. Wang et al. [231] investigated a novel mRNA delivery platform in which lipid nanoparticles were coated with bacterial outer membrane vesicles (OMVs). Using OMVs derived from Escherichia coli as a model, they coated LNPs carrying mRNA encoding the dengue virus (DENV) E80 protein to generate OMV-LNPmRNA. This formulation markedly increased DENV-neutralizing antibody titers in mice, with antibody levels persisting for at least two months post-immunization. OMV-LNPmRNA also significantly promoted the formation of germinal center B cells and memory B cells, indicating the induction of long-lasting immune memory. Such durable immunity provides a promising new approach for addressing complex pathogens and long-term immunization needs. Vadovics et al. [232] examined four distinct LNP formulations (LNP Q, LNP H, LNP Y, LNP W) to evaluate the influence of PEG-lipid ratios and phospholipid types on adjuvant activity. Among these, LNP H (0.5% PEG, DSPC phospholipid) demonstrated the strongest humoral immune response, inducing higher binding antibody levels, superior viral neutralization, and more robust memory B-cell and long-lived plasma cell responses. These findings show that LNP H elicits strong antibody responses and durable immune memory. Modulating PEG-lipid content and phospholipid composition allows fine-tuning of the strength and quality of immune responses elicited by mRNA vaccines, offering important scientific guidance for designing next-generation LNPs to optimize vaccine efficacy.

#### Long-term immunogenicity, safety concerns, and future research priorities

Although LNPs show promising potential in mRNA delivery, current research still has limitations that warrant deeper investigation. One major gap is the limited understanding of the long-term immunogenicity of LNPs. Most existing studies focus on short-term immune responses and the immediate effectiveness of vaccines, leaving significant uncertainty regarding chronic effects and long-term safety, particularly for therapeutic applications requiring repeated dosing, such as those for chronic or genetic diseases. This underscores the need for systematic long-term studies to elucidate immune tolerance, chronic inflammation, and the cumulative impact of repeated administration.

In addition, safety concerns associated with LNP immunogenicity remain highly important. Risks such as anaphylaxis, complement activation-related pseudoallergy (CARPA), and potential autoimmune responses represent critical challenges in LNP-based delivery [233]. Although these adverse events have been widely documented, their underlying mechanisms are not yet fully understood, and comprehensive strategies to mitigate these risks are lacking. Therefore, advancing mechanistic research and developing safer LNP formulations are essential to minimize adverse effects while maintaining therapeutic efficacy.

## Future prospects and emerging innovations

### Optimization of mRNA sequence engineering and delivery platforms

**Fig. 4:**
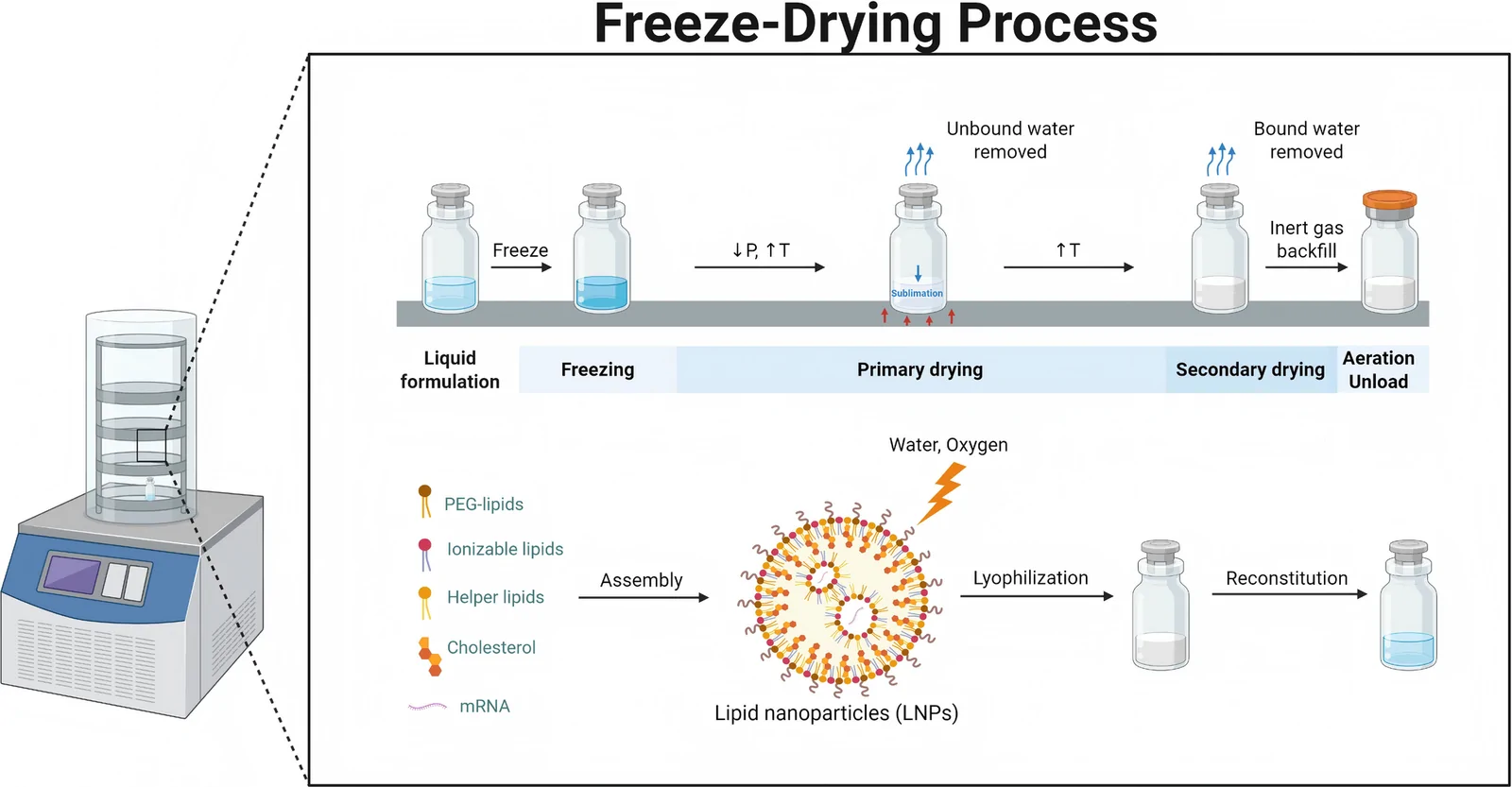
Freeze-drying process at macro and micro scales. This illustration outlines the freeze-drying process for lipid nanoparticle (LNP)–mRNA formulations across macro and micro scales. The schematic includes a freeze-dryer apparatus on the left and a detailed flowchart depicting the key stages of the process: Freezing, primary drying, secondary drying, and reconstitution. The micro-scale assembly of LNPs is shown, with individual lipid components (PEG-lipids, ionizable lipids, helper lipids, cholesterol) interacting with mRNA. The process includes water removal steps—Unbound water is removed during primary drying, followed by the removal of bound water during secondary drying. The final stage involves reconstitution of the lyophilized LNP–mRNA formulation

To begin with,optimizing mRNA sequence engineering and delivery systems is central, with the goals of improving thermal stability and safety while reducing adverse reactions.Existing mRNA-LNP vaccines (e.g., mRNA-1273 and BNT162b2) require storage at −20 °C and −60 °C to −80 °C, respectively, posing substantial cold-chain challenges in resource-limited settings.mRNA hydrolysis, driven by residual water molecules triggering phosphodiester cleavage, is a primary cause of instability in mRNA-LNP vaccines [234]. To address this, lyophilization (freeze-drying) removes water via sublimation, effectively slowing degradation and offering a feasible path toward ambient-temperature storage by enhancing stability and extending shelf life (Fig. 4) [235]. However, the process must preserve key physicochemical attributes, including particle size distribution, encapsulation efficiency, mRNA integrity, and immunogenicity. For instance, Muramatsu et al. demonstrated that mRNA-LNPs formulated in a Tris-sucrose-maltose buffer remained stable for 12 weeks at room temperature and 24 weeks at 4 °C [236]. Ai et al. reported a lyophilized Omicron vaccine stable for over 6 months at 25 °C while maintaining robust immunogenicity. Clinically, Moderna’s Phase III CMV vaccine (mRNA-1647, NCT05085366) has a lyophilized presentation stable for up to 18 months at 5 °C. Looking forward, alternative drying technologies such as spray-drying, spray freeze-drying, and thin-film freeze-drying are being explored to further improve the stability and scalability of dried mRNA-LNP formulations.In addition, alternative drying technologies, such as spray-drying, spray freeze-drying, thin-film freeze-drying, and supercritical spray-drying, are being explored to further improve stability and scalability [237].

Artificial intelligence is opening new avenues for rational and automated mRNA design. Wayment-Steele et al. crowdsourced AI models to predict RNA degradation rates, highlighting deep learning’s potential for stabilizing RNA [238]. Leppek et al. developed the In-line-seq platform to map sequence–structure rules that minimize hydrolytic cleavage, thereby improving mRNA stability and translational efficiency. Zhang et al. introduced the AI design algorithm LinearDesign, which markedly enhanced immunogenicity of mRNA vaccines against COVID-19 and varicella-zoster virus (VZV), with up to 128-fold higher neutralizing titers in mice [239]. Further studies demonstrated that optimizing mRNA structural features can regulate functional half-life and protein expression, providing additional principles for the rational engineering of next-generation mRNA vaccines [240].

With respect to safety, unwanted immune reactivity remains a key concern. Sensitizing constituents in vaccines, such as glycoprotein peptidase fragments (amino acids 437–508) and excipients (sucrose, polyethylene glycol), may trigger IgE-mediated allergic inflammation. Such reactions can drive release of histamine, prostaglandins, leukotrienes, proteases, and pro-inflammatory cytokines, leading to rash, laryngeal edema, hypotension, or even cardiovascular collapse [241]. Although nucleoside modifications can substantially reduce the innate immunostimulatory properties of mRNA and improve LNP immunogenic profiles, high doses or specific ionizable lipids can still provoke immune activation and cytotoxicity, reflecting dependencies on lipid composition and payload.

### Advancing mechanistic understanding of immune activation and adjuvant strategies

Furthermore, Deeper mechanistic insight into innate and adaptive immune activation, together with innovative adjuvant strategies, is essential for tailoring responses and achieving optimal efficacy across pathogen classes.

#### Computationally guided immuno-design

Traditional vaccine development has been largely empirical, time-consuming, and resource-intensive, with limitations in addressing diverse pathogens. Advances in immunoinformatics and systems biology are ushering in a computation-driven era that dissects immune recognition at the molecular level and accelerates the creation of cross-protective vaccines. Researchers are actively pursuing multiepitope, broad-spectrum designs. For example, immunoinformatics pipelines have been used to construct multiepitope mRNA candidates against monkeypox, smallpox, and vaccinia, illustrating how in silico design can expand antigen coverage and specificity [242].

#### Overcoming organ-specific delivery barriers

Conventional LNPs display strong hepatic tropism, largely due to ApoE-mediated uptake by Kupffer cells and liver sinusoidal endothelial cells. While advantageous for liver-targeted indications, this limits efficient extrahepatic delivery. Selective Organ Targeting (SORT) strategies are under intensive study. Lipid-component engineering, such as using 5A2-SC8 backbones and adding cationic DOTAP, reduces ApoE binding and improves targeting of lung and spleen. Tuning surface charge can further lower hepatic uptake, and ligand conjugation (antibodies, peptides, polysaccharides) has markedly improved extrahepatic delivery efficiencies [243].

#### Route of administration and particle design

Intranasal and intradermal routes engage local immune tissues directly. Nanoparticles of 20–200 nm are more readily internalized by dendritic cells, amplifying antigen presentation and immune activation. Although efficient extrahepatic mRNA delivery remains a frontier challenge, innovations in lipid chemistry and ligand-guided targeting have yielded breakthroughs for the lung, tumors, and even the central nervous system. Future work should focus on raising targeting precision and safety while developing scalable, well-controlled manufacturing, thereby enabling broader clinical translation.

### AI-enabled and high-throughput approaches for antigen discovery and vector optimization

In addition, Convergence of artificial intelligence, bioinformatics, and high-throughput screening to accelerate antigen discovery and delivery-vector optimization.

#### AI-enabled antigen and vector design

AI has become a central driver of antigen identification and structural optimization. Structure-prediction tools exemplified by AlphaFold improve our understanding of antigen 3D conformations and immune recognition, supporting epitope selection and immunogenicity prediction [244]. In practice, Moderna and BioNTech have deployed machine-learning models end-to-end—mining viral sequence space to prioritize immunogenic regions, shorten design cycles, and broaden coverage. Platforms such as Vaxign-ML and deep-learning epitope predictors are expanding target discovery for influenza, HIV, and other viruses [245].

#### Combinatorial chemistry meets AI

High-throughput combinatorial platforms established in 2023 enabled the synthesis and screening of biodegradable ionizable-lipid libraries (e.g., 3-component reactions) for inhalable mRNA and CRISPR–Cas9 delivery [246]. In 2024, an improved 4-component reaction toolkit identified lipid surpassing DLin-MC3-DMA in transfection across multiple tissues and immune cell types [247]. The AGILE (AI-Guided Ionizable Lipid Engineering) platform further integrates deep learning with combinatorial chemistry to rapidly predict properties and tailor designs to specific cell types. Complementary efforts employing LightGBM and other models have screened tens of millions of candidates to discover high-performance lipids. Using AI tools like graph neural networks and Transformer-based predictors, researchers now accurately estimate key LNP parameters (e.g., pKa, transfection efficiency) and, more critically, design novel ionizable lipids. These lipids surpass DLin-MC3-DMA in function and show translatable pulmonary delivery across species [200].

#### Multimodal modeling and experimental surrogates

The GENESIS288 system integrates multimodal analytics to simulate lipid–cargo interactions, membrane-fusion dynamics, and biodistribution at atomic resolution, complementing conventional molecular simulations [248]. On the spatial biology front, single-cell and spatial transcriptomics reveal heterogeneity in cell types and receptor repertoires within target tissues, guiding precise surface functionalization. In tumors, CD206⁺ tumor-associated macrophages and αvβ3-rich dendritic cells emerge as specific targets; mannose-modified LNPs selectively deliver to CD206⁺ TAMs/DCs, while RGD-conjugated LNPs preferentially bind αvβ3-high DCs. Organ-on-a-chip models (e.g., BBB, hepatic sinusoids, alveoli) provide biomimetic systems for in vitro LNP screening and mechanism studies, supporting expansion toward non-immune organs and cross-disease applications [249].

Computer-aided design (CAD) integrated with AI is reshaping LNP formulation paradigms in vast, intertwined chemical spaces by linking molecular features to performance for rapid triage and rational optimization. ANN- and DOE-based frameworks have yielded efficient mRNA-LNP vaccines, and machine-learning models have predicted in vivo delivery efficiency for siRNA–LNPs. AGILE-like platforms identify experimentally validated, high-performance molecules from thousands of candidates, enabling customized targeting strategies. Going forward, open, high-quality datasets and multimodal training frameworks will be pivotal to fully realize AI’s potential across antigen discovery, lipid generation, and organ-level targeting.

### Expansion of application domains and advancement of personalized immunotherapy

Moreover,Broadening application domains and advancing personalized immunotherapy to tackle bacteria, parasites, and polymicrobial infections:

In individualized oncology, whole-exome or transcriptome analyses of patient tumor samples reveal patient-specific somatic mutations. Algorithms such as NetMHCpan predict neoepitopes arising from these mutations [250]. LNPs then deliver mRNAs encoding the neoantigens, which are presented via MHC class I to induce CD8⁺ cytotoxic T-cell activation and selective tumor clearance. By anchoring mRNA vaccine delivery, LNPs are catalyzing a paradigm shift in cancer care—from nonspecific cytotoxicity toward precision immune activation.

Concurrently, LNP platforms are being continuously optimized within AI-driven “design–screen–validate” cycles, integrating advances in nanomaterials engineering, immunology, and cell biology [251]. Engineered LNPs are multifunctional, programmable, and highly reliable, supporting precision medicine—especially targeted delivery and disease-specific intervention. This cross-disciplinary integration is evolving LNPs from simple vaccine carriers into broad therapeutic platforms, with applications now spanning cancer immunotherapy, autoimmune disorders, and gene editing (e.g., CRISPR/Cas9 mRNA). New strategies, such as ligand modification, stimuli-responsive release, and deep integration with AI models, are significantly enhancing delivery efficiency and biosafety, expediting clinical translation.

At present, lipid-nanoparticle–based RNA vaccines are being clinically tested against multiple pathogen classes (bacteria, parasites, viruses), with fungi representing a remaining gap. As approvals accumulate, research foci are expanding to bacterial, fungal, and protozoan targets. Incorporating suitable adjuvants can maintain immunogenicity while reducing ionizable-lipid doses, thereby lowering reactogenicity and improving safety. For instance, embedding 5 mol% of a TLR7 agonist within the lipid architecture to form a pentalipid LNP markedly increased humoral and cellular responses to an mRNA-LNP SARS-CoV-2 vaccine—an approach especially valuable for multivalent or pan-pathogen constructs comprising multiple antigen-encoding mRNAs [252], where dose-limiting toxicity must be mitigated.

Finally, Large-scale, long-duration clinical studies, integrated with systematic real-world data surveillance, are essential for evaluating durability of immunity, breadth of cross-protection, and rare adverse events. Such evidence will elucidate the long-term dynamics of immune responses and inform optimization of vaccination strategies and risk management. With sustained cross-disciplinary collaboration and technological innovation, mRNA vaccines are poised to retain a central role in the control of current and future global infectious diseases.

## Conclusions

This Review integrates evidence from sequence engineering, lipid nanoparticles, manufacturing and regulation, and advances an integrative framework: against the backdrop of established clinical feasibility, stability and access remain the key constraints on global deployment. We relate in-vivo and in-vitro critical quality attributes to long-term storage and distribution requirements, indicating that joint optimization of RNA sequences and lipid components, coupled with lyophilization-ready, cold-chain-sparing process improvements, provides a clear technical path toward next-generation products that are thermostable and low-dose. Next, within a harmonized evaluation framework that combines standardized metrics with real-world data, there is a need to systematically evaluate the duration of immune protection and safety; meanwhile, in combining emerging RNA formats such as saRNA and circRNA with adjuvanted LNPs, artificial intelligence brings sequence design, lipid screening and process parameters into a single data framework and, leveraging interpretable multiscale models and active experimental design, shortens optimization cycles and accelerates clinical translation while expanding global access.

## Data Availability

Not applicable.
